# *Opisthorchis felineus* infection, risks, and morbidity in rural Western Siberia, Russian Federation

**DOI:** 10.1371/journal.pntd.0008421

**Published:** 2020-06-29

**Authors:** Olga S. Fedorova, Marina M. Fedotova, Olga I. Zvonareva, Sofia V. Mazeina, Yulia V. Kovshirina, Tatiana S. Sokolova, Ekaterina A. Golovach, Anna E. Kovshirina, Uliana V. Konovalova, Ivan L. Kolomeets, Sergey S. Gutor, Vyacheslav A. Petrov, Jan Hattendorf, Ludmila M. Ogorodova, Peter Odermatt

**Affiliations:** 1 Department of Faculty Pediatrics, Federal State Budget Educational Institution of Higher Education, Siberian State Medical University, Ministry of Healthcare of Russian Federation, Tomsk, Russian Federation; 2 Department of Health, Ethics and Society, Maastricht University, Maastricht, the Netherlands; 3 Central Research laboratory, Federal State Budget Educational Institution of Higher Education, Siberian State Medical University, Ministry of Healthcare of Russian Federation, Tomsk, Russian Federation; 4 Shegarskiy Regional Hospital, Tomsk Oblast, Russian Federation; 5 Department of Infectious diseases and Epidemiology, Federal State Budget Educational Institution of Higher Education, Siberian State Medical University, Ministry of Healthcare of Russian Federation, Tomsk, Russian Federation; 6 Department of Epidemiology and Public Health, Swiss Tropical and Public Health Institute, Basel, Switzerland; 7 University of Basel, Basel, Switzerland; Seoul National University College of Medicine, REPUBLIC OF KOREA

## Abstract

**Background:**

The liver fluke, *Opisthorchis felineus*, is widely distributed throughout Europe and large parts of the Russian Federation. In Western Siberia, information about opisthorchiasis is lacking although infection may lead to severe liver and bile duct diseases. We aimed to assess the current prevalence of *O*. *felineus* infection along with associated risk factors and morbidity in rural Western Siberia.

**Methods:**

We conducted a community-based, cross-sectional study in the rural Shegarskiy district, Tomsk Oblast, Russian Federation. All household members (≥ 7 years) present on the survey day were enrolled (n = 600). Two stool samples per person were examined for helminth eggs, using PARASEP (DiaSys Ltd, UK). The number of eggs per gram (EPG) of feces was recorded. Each study participant was interviewed to determine risk factors, using a pre-tested questionnaire. An abdominal ultrasonography examination of liver and bile ducts was performed with a mobile, high resolution ultrasound device. In total, 488 persons completed assessments (two stool samples, completed questionnaires); of those, 436 individuals had an ultrasonography (US) examination.

**Results:**

We observed a prevalence of *O*. *felineus* infection of 60.2%. Significant risk factors for infection were the consumption of river fish (odds ratio from adjusted analysis [aOR] 2.4, 95% CI 1.52–3.95, p<0.001), particularly stock fish (OR from multivariable analysis [mOR] 3.2, 95% CI 2.63–3.80, p<0.001), smoked fish (mOR 1.5, 95% CI 1.24–1.72, p<0.001), frozen fish (mOR 1.6, 95% CI 1.29–2.02, p<0.001), and raw fish (mOR 1.4, 95% CI 1.05–1.84, p = 0.02); and fishing activities (mOR 1.2, 95% CI 1.03–1.43, p = 0.019). Women had a higher risk of infection than men. Infection was associated positively with age and negatively with socio-economic status. The respondents’ general awareness of opisthorchiasis was quite high (93.2%), but their knowledge about infection transmission and prevention was insufficient. Children aged 7–18 years old had a lower level of awareness compared to adults. The abdominal ultrasonography results demonstrated a strong association between *O*. *felineus* infection and gallbladder stones (mOR 2.8, 95% CI 1.33–6.04, p = 0.007) and periductal fibrosis of intrahepatic bile ducts (mOR 1.9, 95% CI 1.08–3.46, p = 0.026).

**Conclusion:**

*O*. *felineus* infection is highly prevalent in rural regions of Western Siberia, and associated with severe hepatobiliary pathology. Identified risk factors will be used to develop a comprehensive targeted *O*. *felineus* infection control program.

## Author summary

*Opisthorchis felineus* is a trematode (liver fluke) that infects the hepatobiliary systems of humans in endemic regions. The infection may lead to severe liver and bile duct diseases, making it a public health issue in endemic communities like Western Siberia. Yet, little information is available from this region about its epidemiology. We aimed to assess the current prevalence of *O*. *felineus* infection along with associated risk factors and morbidity in one district in rural Western Siberia. We conducted a cross-sectional community-based study in the Shegarskiy district, Tomsk Oblast, Russian Federation. All household members (≥ 7 years) present on the survey day were enrolled (n = 600). Two stool samples per person were examined for helminth eggs. We interviewed study participants to determine risk factors using a questionnaire. An abdominal ultrasonography examination was performed to assess liver and bile duct pathology. In total, 488 persons completed assessments. We found a high *O*. *felineus* infection prevalence of 60.2%. Women and older study participants had a higher risk of infection. The consumption of river fish, particularly stock, smoked, frozen and raw fish; and fishing activities increased the risk of infection. Study participants with a higher socio-economic status had a lower risk of infection. The ultrasonography showed that participants with infection had an increased risk of gallbladder stones and bile duct pathologies. Our study provides valuable information about *Opisthorchis* infection prevalence, its risk factors and morbidity in rural Siberia. The results will be useful for developing a multidisciplinary, community-based control program against *Opisthorchis* infection in endemic regions.

## Introduction

The infection caused by the liver fluke, *Opisthorchis felineus*, belongs to the group of neglected tropical diseases (NTDs), but is widely distributed throughout the northern hemisphere [1]. *O*. *felineus* infection is endemic in Southern and Eastern Europe and in large parts of the Russian Federation, particularly in Western Siberia [2–9]. *O*. *felineus* is considered to be zoonotic [1]. Most infections with *O*. *felineus* are asymptomatic [10]. Chronic *O*. *felineus* infection may lead to any of several severe liver and bile duct diseases, such as cholecystitis, cholangitis, and periductal fibrosis. In addition, there is evidence that *O*. *felineus* is a risk factor for cholangiocarcinoma (CCA), a fatal bile duct cancer [3,4,11]. Recent studies indicate that a chronic *O*. *felineus* infection modifies immune reactivity [12].

*O*. *felineus*, a close relative of the Asian liver fluke, *Opisthorchis viverrini*, has a complex life cycle that includes two freshwater intermediate hosts; namely, a mollusk of the genus *Bithynia* and the fish species of the family *Cyprinidae*, which act as first and second intermediate hosts, respectively [1,13]. Humans acquire an infection through the consumption of raw or insufficiently cooked cyprinoid fish [14–16]. A wide range of mammals such as cats, dogs, foxes, and bears, [1,13] are known reservoir hosts of *O*. *felineus* and are responsible for transmitting the fluke to *Bithynia* snails [14,17].

Human infection with *O*. *felineus* was first described in 1891 by Konstantin Vinogradov, from Tomsk Imperial University. While conducting an autopsy of a person from Siberia, who had suffered from jaundice, he detected *Opisthorchis* eggs in the bile of the gallbladder [18,19]. Today, according to official medical statistics, the incidence of *O*. *felineus* infection is 188.8 cases per 100,000 population/year in Tomsk Oblast, through this is likely an underestimation due to underreporting [3]. There is, however, a lack of information about *O*. *felineus* infection and its associated morbidity and risk factors in endemic communities.

This study aimed to assess the current prevalence of *O*. *felineus* infection, its risk factors and morbidity in rural Western Siberia. A community-based, cross-sectional study was conducted in the rural Shegarskiy district, Tomsk Oblast, Russian Federation.

## Materials and methods

### Ethical consideration

All study procedures complied with the ethical standards of the Helsinki Declaration of the World Medical Association. The study protocol was approved by the Ethical Committee of the Siberian State Medical University (SibMed), Tomsk (No. 4815, 27.06.2016). Written informed consent was obtained from adult and adolescent (14 years and older) study participants. Parents or legal guardians of children under 14 years signed the informed consent forms.

### Study setting

**Fig 1:**
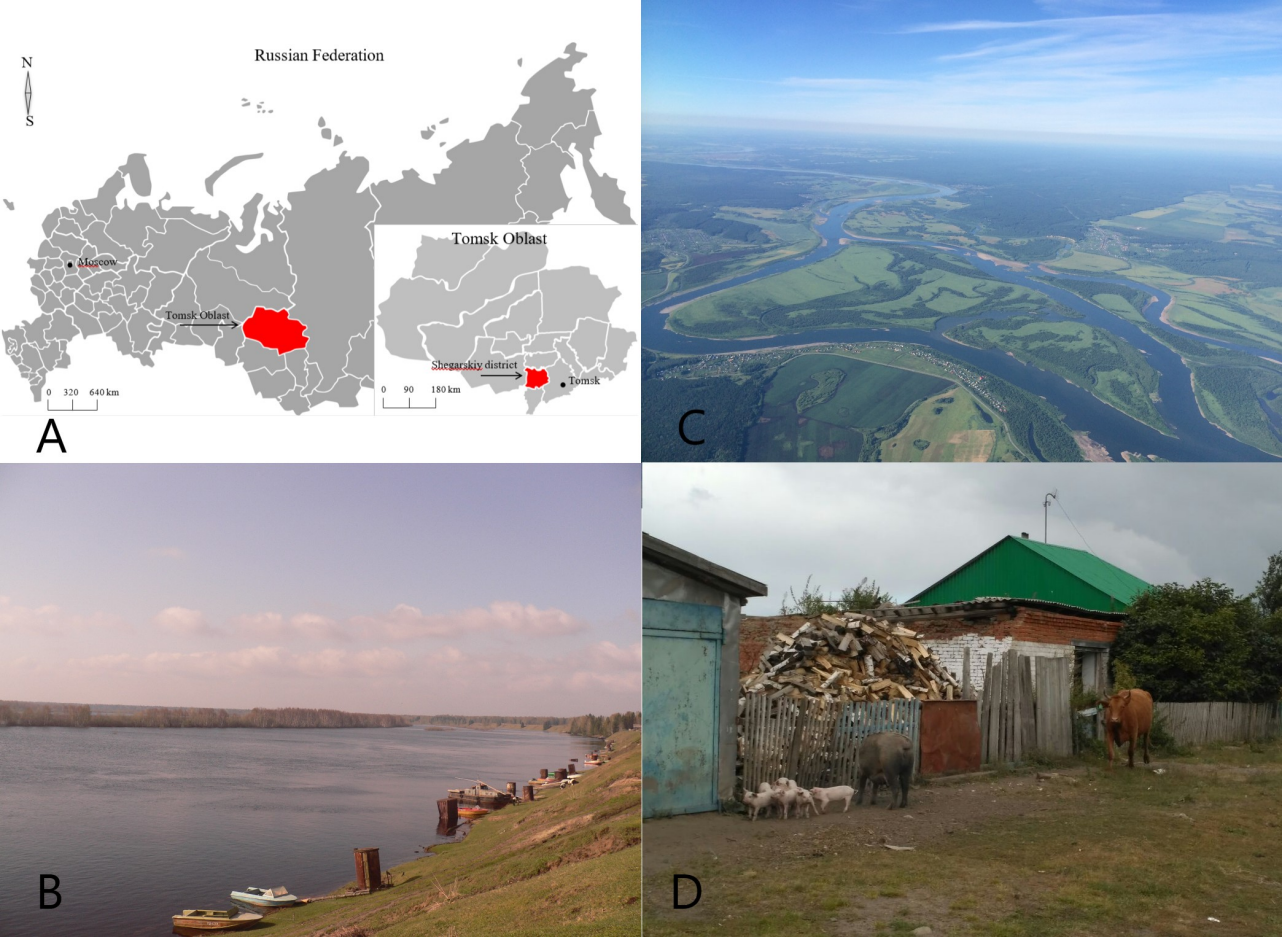
Study location. A. Study setting: map of Russian Federation, map of Tomsk Oblast, B. River Ob, C. Areal picture of the study location with Ob river, D. Typical village household, Shegarskiy district, Tomsk Oblast. Microsoft Office Visio 2007.

The study was conducted in the Shegarskiy district of Tomsk Oblast, in the Siberian Federal District, Western Siberia (Fig 1). The district center, Melnikovo, is located about 100 km west of Tomsk city in the middle of the Russian Federation (56^o^34’ N, 84^o^ 5’ E). Tomsk city is the capital of the Tomsk Oblast.

The rural Shegarskiy district is 5 029 km^2^ and has a population of approx. 19,200 [20]. Melnikovo accounts for 41.3% of the district's total population. There are 36 small villages in the Shegarskiy district. The two main rivers are the Ob (East) and the Shegarka (West). The Ob is a major river in Western Siberia, and the seventh-longest river in the world. Among Russian rivers, it has the largest basin territory. The climate is continental with an average temperature of −23°C in January and +20°C in July [21].

The people of Shegarskiy district have a typical rural/farmer lifestyle. Life expectancy in the district is approximately 71.7 years [21]. Farming, transport, and production of wood, construction materials, and food are the main sources of revenue for the population. With a rich network of rivers and lakes, the Shegarskiy district has high fish densities. Previous research has shown the importance of river fish to the local population in terms of nutrition, food culture and community life [22].

The health care infrastructure consists of the Shegarskiy Central District Hospital in Melnikovo, four general practice offices and eleven small local medical units, with a nurse and / or an obstetrician nurse in the bigger villages. In 2010, the prevalence of *O*. *felineus* infection among children 7–11 years old was 20.8% in Shegarskiy district [12].

### Study population and design

A cross-sectional study was conducted in the Shegarskiy district, Tomsk Oblast, between 1 July 2016 and 30 April 2017. A random cluster sample with sampling probability proportional to village population size with replacement was drawn. To reach the anticipated sample size of 600, we drawn with replacement 30 times a village and the number of persons to be sampled in each village was calculated as the number of time the village has been selected multiplied by 20. In the selected villages, households were randomly selected based on a list of all households. The inclusion of households continued until the number of enrolled study participants corresponded to the calculated sample size.

**Fig 2:**
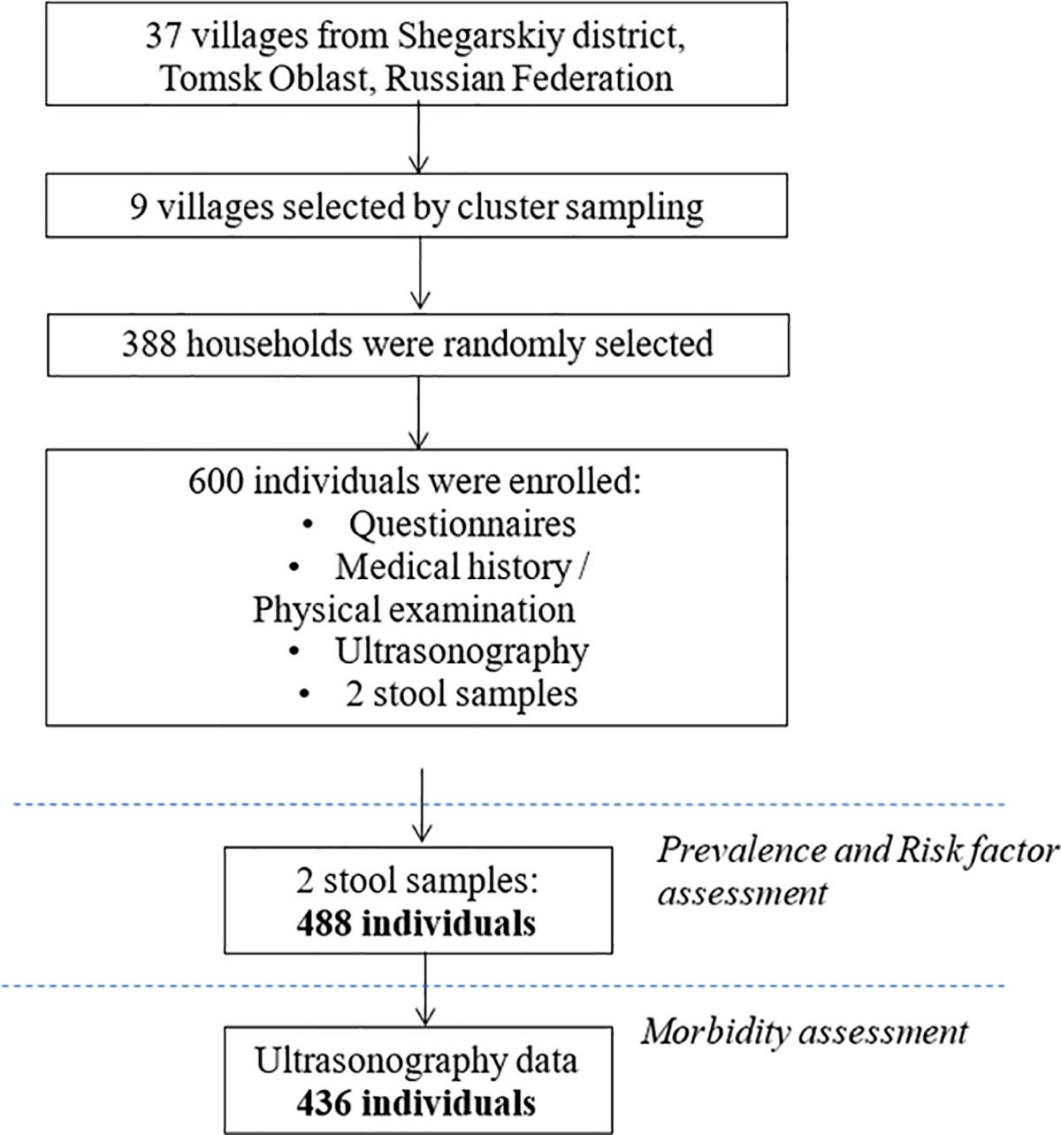
Study diagram.

Using the cluster approach, nine of the 37 villages were selected, namely Melnikovo, Kargala, Voznesenka, Batkat, Pobeda, Monostyrka, Malobragino, Novoilyinka, and Voronovka. For each village, the list of households was obtained and the households were randomly selected. All household members (aged seven years and older) present on the survey day were enrolled. Each enrolled person underwent an individual interview and a clinical examination. In addition, they provided two fecal samples on consecutive days. Finally, each participant was submitted to a comprehensive abdominal ultrasonography assessment (Fig 2). All interviewers and clinicians were trained in order to optimize assessment procedures. Laboratory technician and radiologist were unaware of the results of the other assessments. The sample size was determined based on an expected prevalence of 20%, defined as one half length of the 95% confidence interval, of 5 percentage-points and a design effect of 2 to account for cluster sampling. The calculated sample size of 492 was inflated by 20% to account for refusals and incompletes.

### Field and laboratory procedures

#### Interviews

The individual questionnaires assessed potential risk factors for *O*. *felineus* infection and related morbidity. It collected data about sociodemographic factors (age, sex, gender, education, profession, socio-economic status [SES]); customary practices of raw fish consumption; personal hygiene; and knowledge, attitude and awareness of *O*. *felineus* infection, transmission and disease. The questionnaire was developed based on the insights gained from in-depth interviews conducted during an earlier qualitative study [22]. The questionnaire was developed in English and translated into Russian. The questionnaires were administered by trained interviewers. After a pilot test with 15 volunteers, the questionnaire was revised to improve clarity. Detailed information about each household and its sanitary conditions was obtained via a household questionnaire, administered to the head of the household.

#### Physical examination

Study participants underwent a clinical examination by physicians from the SibMed. Height, weight, blood pressure, heart rate and breathing rate measurements were recorded. Physical examination included a general examination of skin, eyes, upper respiratory tract, lymph nodes, bone, and muscular, respiratory, cardiovascular, digestive, and urinary systems. Special attention was paid to the hepatobiliary system. All results were recorded in the case report form.

#### Stool examination

Pre-labeled stool containers in plastic bags were given the study participants, collected on consecutive days, and transported in cooled containers (4°C) to the laboratory at SibMed in Tomsk. There the samples were examined using PARASEP (DiaSys Ltd, UK). Helminth eggs were identified using a microscope with magnification 100X, 100 microscopic fields were investigated per sample. The number of eggs was counted and recorded for each helminth species separately and the infection intensity (number of eggs per gram stool (EPG)) was calculated.

#### Ultrasonography of the liver and bile ducts

Ultrasonography (US) of the liver and bile ducts was performed by a mobile, high resolution ultrasound device (Shenzhen Mindray Bio-Medical Electronics, Co, Ltd). An Ultrasound Transducer Type: portable ultrasound scanner "Mindray M7", with a color doppler system was used. A convex transducer with an operating frequency of 3.5 MHz and a linear transducer with an operating frequency of 7.5 MHz was used for 3D imaging. One radiologist performed all US examinations. The following indicators were assessed: height of the left liver lobe, diameter of the aorta, pattern of liver parenchyma, gallbladder size, abnormalities in the gallbladder wall and bile ducts, presence of sludge and stones, enlargement of the extrahepatic bile ducts, presence of mass in the liver, and signs of fatty liver [23,24].

Gallbladders were characterized by a normal length of 30–70 mm in children and 100 mm in adults; thickening of the gallbladder wall was detected if more than 3 mm. Extra-hepatic bile ducts (common hepatic duct and common bile duct) were considered as expanded if they had a diameter of 7 mm or more. Respecting the criteria of the periductal fibrosis grade used in previous epidemiological studies in opisthorchiasis-endemic areas, the following grades were established: Grade 0—no echoes in any segment of the liver, Grade 1—echoes in 1 liver segment, Grade 2—echoes in 2–3 liver segments, Grade 3—echoes in more than 3 liver segments [25].

### Data management and statistical analysis

All data was entered into EpiData v. 3.4 (www.epidata.dk). Statistical analysis was performed in R language (version 3.4.3) [26]. *O*. *felineus* infection intensity was grouped low (0–999 EPG), moderate (1000–9999 EPG), and severe intensities (10,000 and above EPG) [27]. The maximal result of infection intensity from the two stool samples was taken for each participant.

Continuous data were presented in mean and standard deviation, while ranked and nominal variables were presented in percent. Analysis of socioeconomic status (SES) was performed for participants 18 years and older. Status was calculated based on information about a person's education, car ownership, employment status, additional income, and access to central water supply. Principal component (PC) analysis was performed. The first principal component (PC1, 31.1% of explained variance) was used as a marker of a person's SES. According to the numerical value of the first PC, participants were divided into four groups as follows: lowest SES (up to 1^st^ quartile of PC1 value), low income (from 1^st^ to 2^nd^ quartile of PC1 value), moderate income (from 2^nd^ to 3^rd^ quartile of PC1 values), and high income (above 3^rd^ quartile of PC1 values).

Generalized estimating equations for binary outcomes (GEE), geeglm function of geepack R package [28] were used to account for potential correlation within villages. A fully iterated jackknife variance estimator was used to identify associations between *O*. *felineus* infection, risk factors and morbidities. An independent or exchangeable covariance structure for data clusters was chosen by estimating the correlation information criterion for each model. All potential measured risk factors and morbidity were tested in univariate GEE models, adjusting for age and sex as covariates. Based on the results of univariate model testing, all variables with FDR-adjusted p-values less than 0.05 were treated as potential predictors and subsequently included in the multivariable analysis. Multivariate GEE models were applied for risk factor and morbidity predictors separately; each model was tested for collinearity with variance inflation factors (VIF) computation. All predictors with square roots of VIF greater than two were subsequently excluded from the models. The odds ratio (OR) was adjusted for age and sex in the univariable analysis (aOR) and the odds ratios from the multivariable analysis (mOR) were reported with their 95% confidence interval (95% CI) and p-values. A map of the study area was established with Microsoft Office Visio 2007. Map's rendering of the *O*. *felineus* infection prevalence in the studied area was performed with ggplot2 package of the R language. Geodata of Shegarskiy district was obtained from OpenStreetMap.org. www.openstreetmap.org [29].

## Results

### Study population

Overall, 600 participants were enrolled from 388 randomly selected households in nine villages (average number of households per village 43.1, range 11–169; Fig 2). In total, 488 persons completed assessments (two stool samples, completed questionnaires); of those, 436 individuals had an US examination (89.3%).

Among 488 individuals, 30.1% were male and 15.2% were children/adolescents aged 7 to 18 years. Participants’ ages ranged from 7 to 85 years, with a mean age of 46.1 years. The mean number of people per household was 1.5. About half of the participants (52.0%) had completed secondary and/or technical education, while 22.3% had completed higher education. Among adults (≥ 18 years), 42.0% were unemployed; the vast majority were older than 55 years. There were no noteworthy differences in the socioeconomic status between gender groups. More details are provided in Table 1.

**Table 1:** Characteristics of study population (n = 488).

		Total, n	%	Male, n	%	Female, n	%
Total		488	100	147	30.1	341	69.9
Age	Mean (SD)	46.1 (19.7)		44.4 (22.8)		46.9 (18.6)	
	7–11	40	8.2	18	12.2	22	6.5
	12–18	34	7.0	14	9.5	20	5.9
	19–39	92	18.5	23	15.6	69	20.2
	40–59	179	36.7	50	34.0	129	37.8
	60 and above	143	29.3	42	28.6	101	29.6
Village	Batkat	50	10.3	15	10.2	35	10.3
	Kargala	21	4.3	6	4.1	15	4.4
	Malobragino	20	4.1	3	2.0	17	5.0
	Melnikovo	184	37.7	50	34.0	134	39.3
	Monostyrka	21	4.3	9	6.1	12	3.5
	Novoiljinka	20	4.1	5	3.4	15	4.4
	Pobeda	120	24.6	45	30.6	75	22.0
	Voronovka	34	7.0	8	5.4	26	7.6
	Vosnesenka	18	3.7	6	4.1	12	3.5
Education	Incomplete secondary education	71	14.6	24	16.3	47	13.8
	Secondary education	116	23.8	30	20.4	86	25.2
	Technical education	138	28.3	39	26.5	99	29.0
	High education	109	22.3	27	18.4	82	24.0
	Unknown	54	11.1	27	18.4	27	7.9
Employment at moment	Yes	209	42.8	56	38.1	153	44.9
	No	279	57.2	91	61.9	188	55.1
Socioeconomic status	lowest	104	25.1	29	25.2	75	25.0
	low	100	24.1	21	18.3	79	26.3
	moderate	110	26.6	39	33.9	71	23.7
	high	101	24.3	26	22.6	75	25.0

### Prevalence of *Opisthorchis felineus* infection

**Fig 3:**
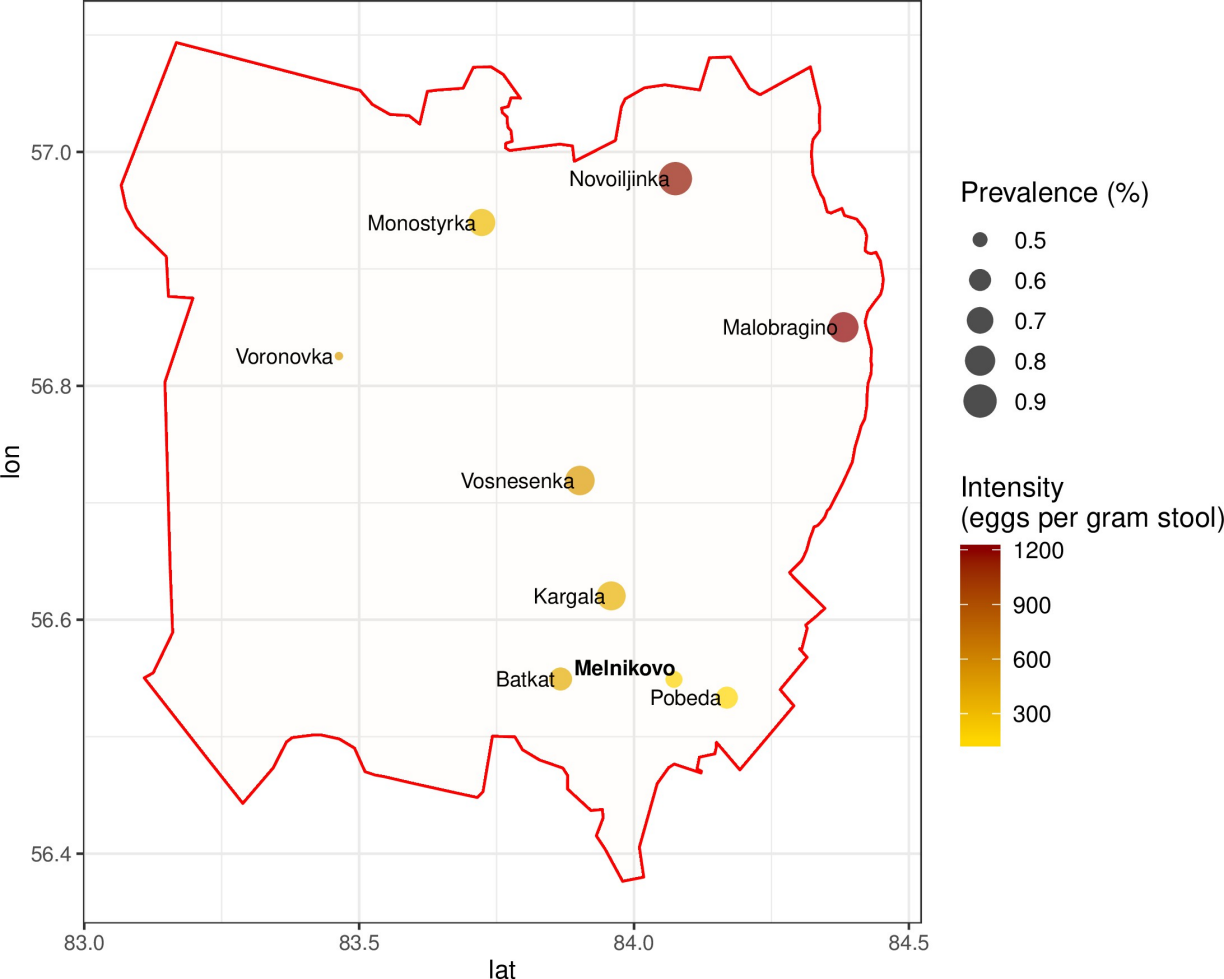
Map of *Opisthorchis felineus* infection prevalence and intensity in Shegarskiy district, Tomsk Oblast. Map's rendering of the *Opisthorchis felineus* infection prevalence in the studied area was performed with ggplot2 package of the R language. Geodata of Shegarskiy district was obtained from OpenStreetMap.org.

The prevalence of any intestinal parasitic infection was 61.5%. *O*. *felineus* was the most prevalent infection, diagnosed in 60.2% of participants (95% CI: 57.3–63.1). *A*. *lumbricoides* (0.4%) and *Giardia intestinalis* (2.7%) were diagnosed in low frequencies. The geographical distribution of *O*. *felineus* infection prevalence is visualized in Fig 3. The highest prevalence rates were detected in Novoiljinka (90.0%) and Malobragino (80.0%), in the northeast of the district. The lowest prevalence was observed in Voronovka (47.1%), in the West of the district. The village is located at a considerable distance from the closest water body. Participants from Melnikovo showed a comparatively low infection prevalence (52.2%). Melnikovo is a semi-urban center in Shegarskiy district. Detailed information on intestinal parasitic infections, stratified by settlement, gender, and age group, is given in S1 Table.

**Fig 4:**
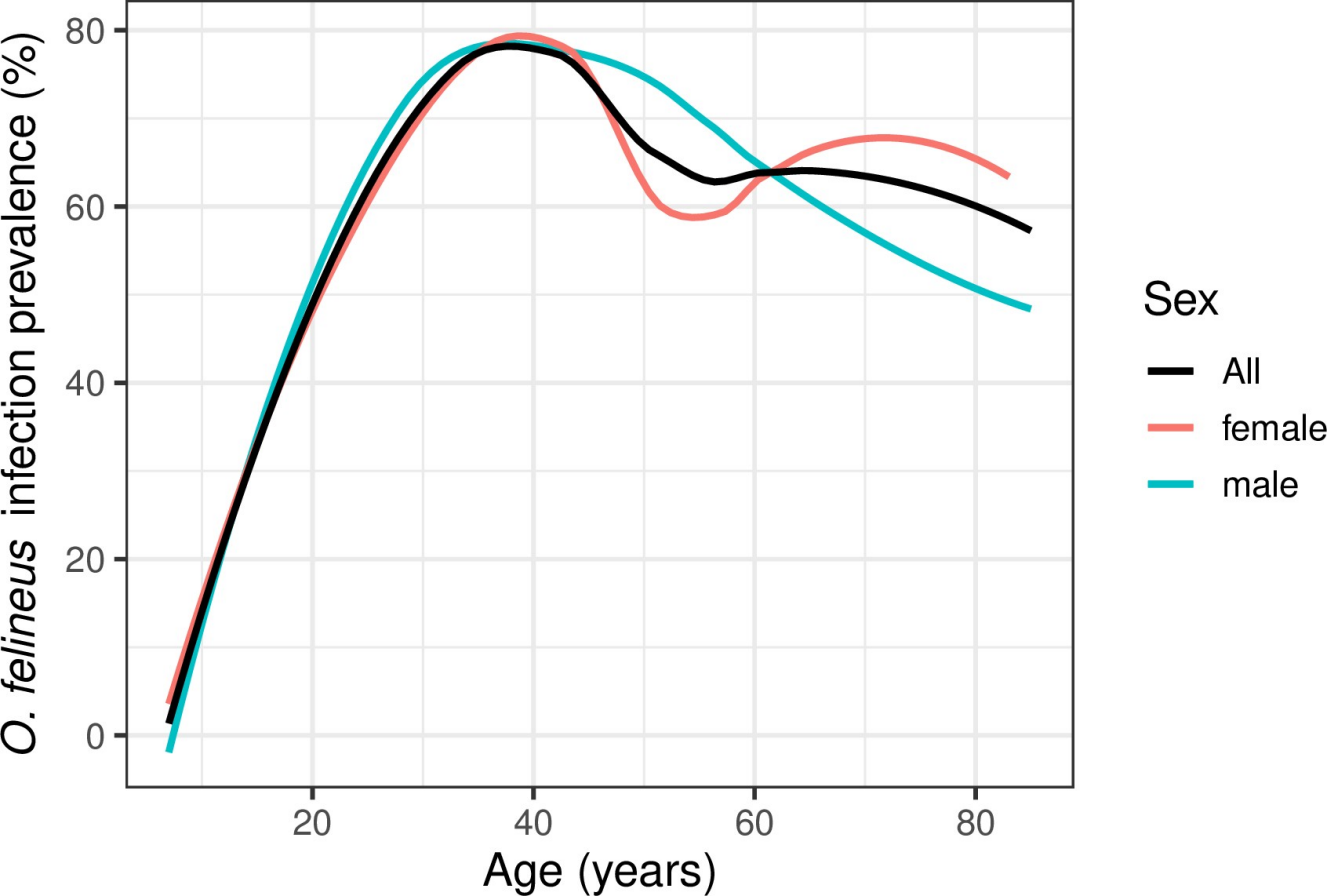
Age-related prevalence of *Opisthorchis felineus* infection in Shegarskiy district, Tomsk Oblast.

Age was a most important factor linked with *O*. *felineus* infection. The prevalence of *O*. *felineus* infection was low among children and gradually increased with age, to a maximum of about 80% infection prevalence among adults around 40 years of age (Fig 4). In study participants aged older than 40 years, infection prevalence decreased to about 50%.

**Fig 5:**
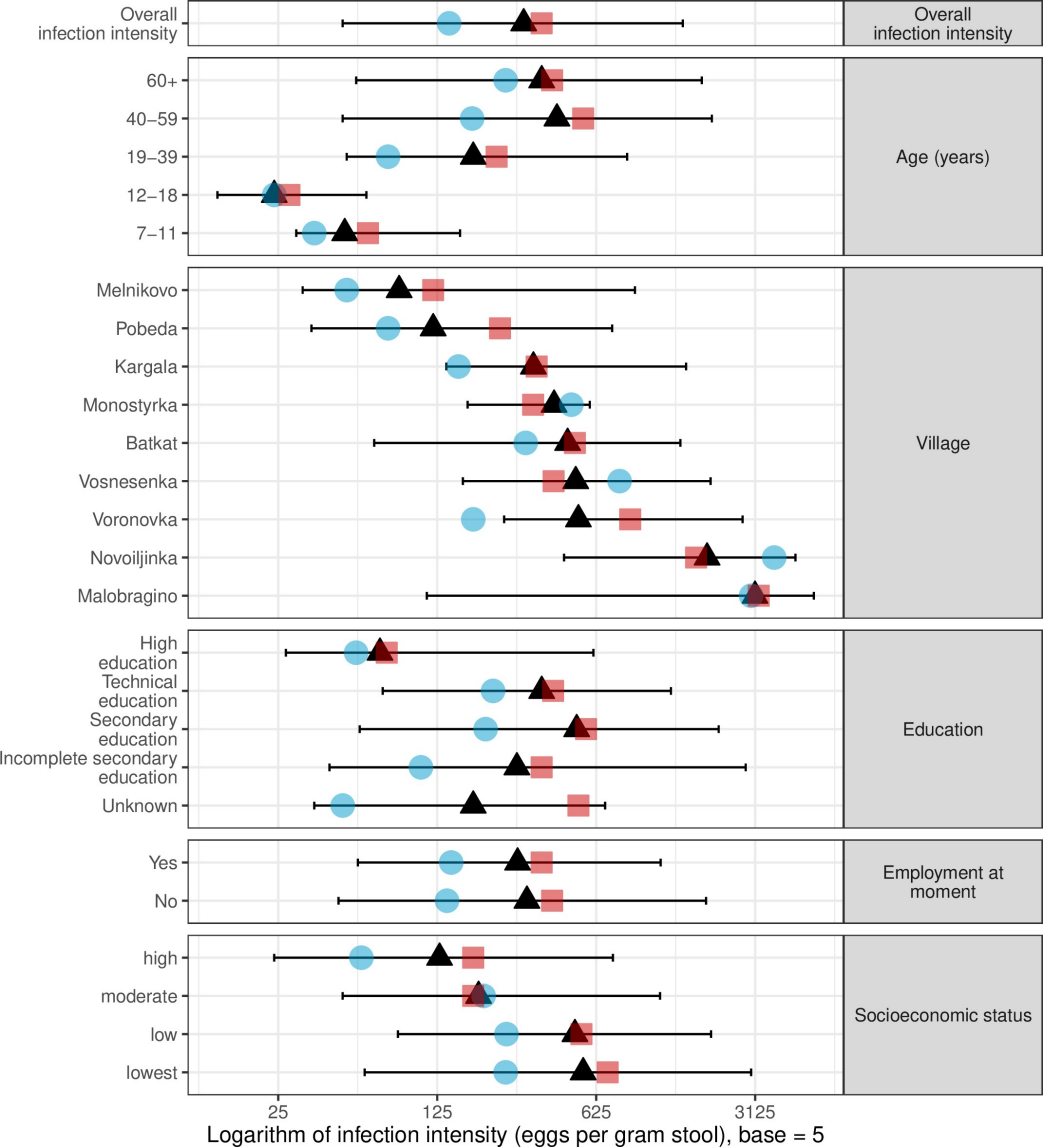
*Opisthorchis felineus* infection intensity in Shegarskiy district stratified by age, residence, education and socio-economic status, Tomsk Oblast. Medians (black triangles), distance between 1^st^ and 3^rd^ quartiles (black bars), medians among males (blue circles), medians among females (red squares).

The *O*. *felineus* infection intensities varied between 2–43,200 EPG, with the overall geometric mean—264 EPG. Among *O*. *felineus-*infected study participants, 70.4% had an infection that was classified as low, 26.5% as moderate, and 3.1% as high-intensity infection. There was no statistical difference (p = 0.33) in infection intensity between male (162 EPG) and female (319 EPG) participants, although males had a lower mean infection intensity than female study participants in all subgroups (Fig 5). Detailed information is provided in the S2 Table.

**Fig 6:**
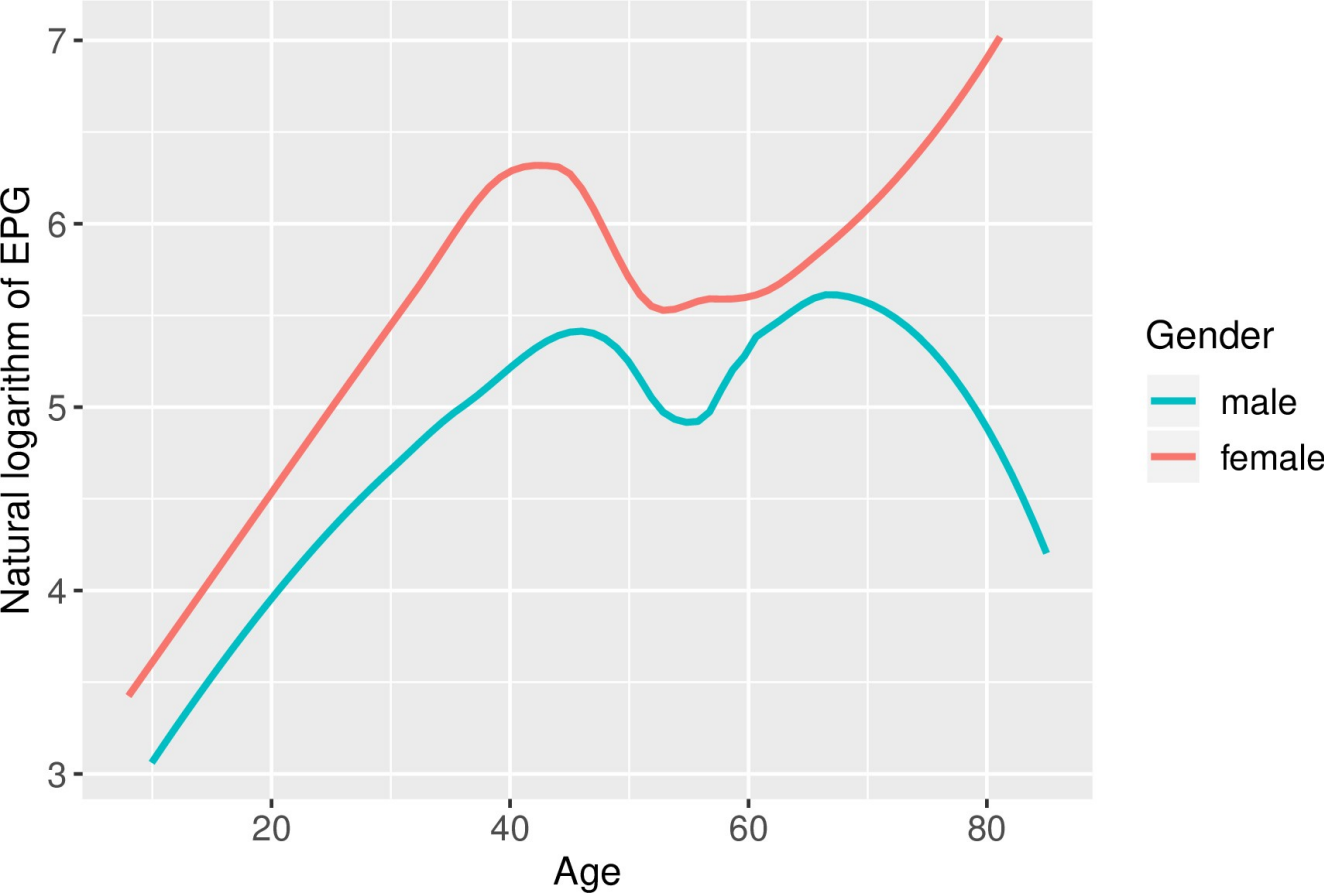
Age-related intensity of *Opisthorchis felineus* infection in Shegarskiy district, Tomsk Oblast (natural logarithm of eggs per gram (EPG)).

**Fig 7:**
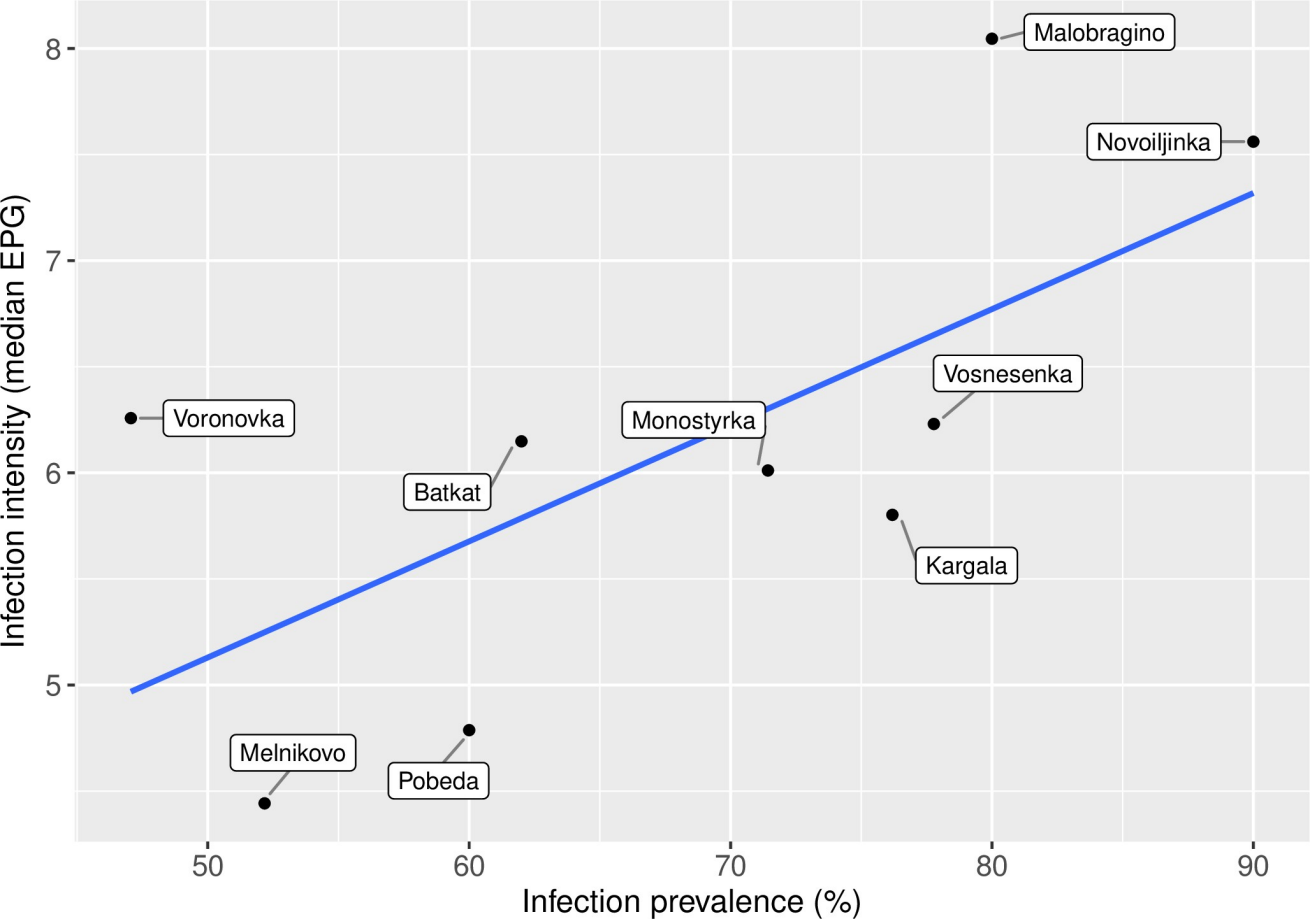
Correlation between *Opisthorchis felineus* infection prevalence and intensity at village level. *Opisthorchis felineus* prevalence and infection intensity assessed as the median infection intensity of positive individuals (natural logarithm, base e) in eggs per gram (EPG) at village level are correlated.

*O*. *felineus* infection intensity was strongly linked with age. The infection intensity in adults was notably higher than in adolescents and children (Figs 5 and 6). At the village level, the mean infection intensity correlated positively with mean infection prevalence (Fig 7). The highest mean infection intensities were detected in Malobragino and Novoilinka (>1000 EPG), while the lowest indicators were in Melnikovo and Pobeda.

### Risk factors for *Opisthorchis felineus* infection

**Fig 8:**
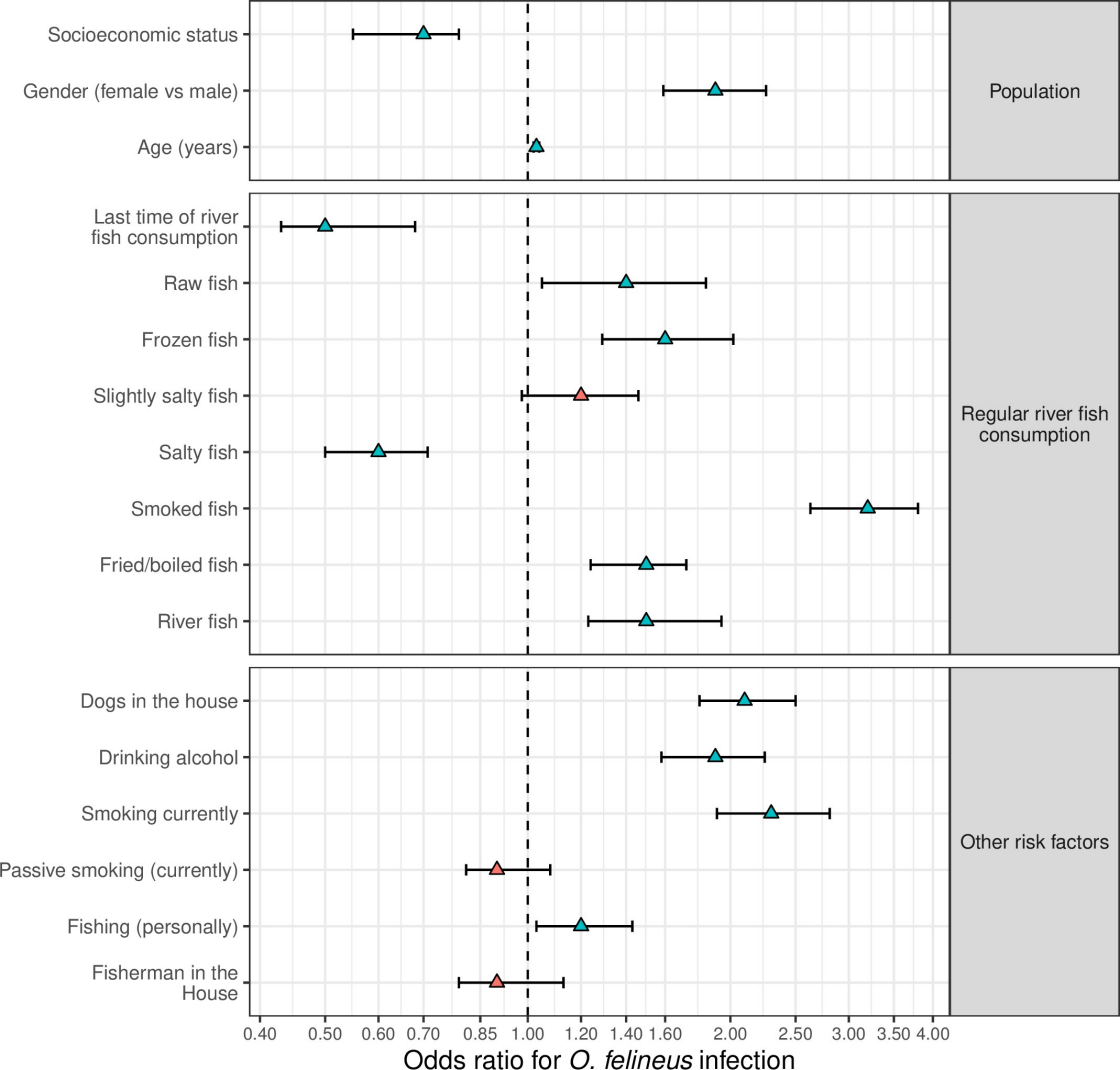
Risk factors of *Opisthorchis felineus* infection. Odds ratio (OR, triangles) and 95% confidence intervals of OR (95% CI, bars) in logarithmic scale (base 5); green and red triangles for statistically significant (p <0.05) and not significant (p ≥0.05) OR; Socioeconomic status (SES) from highest to lowest; last time consuming river fish, from 2 days ago to more than one year ago; for risk factors, yes *versus* no, if not stated otherwise.

In Fig 8 the results of the risk factor analysis are summarized (more details are provided in the S3 Table). Age was positively and significantly associated with an increased risk of *O*. *felineus* infection (mOR = 1.03, 95% CI = 1.02–1.04, p<0.001). Women had a significantly higher risk of *O*. *felineus* infection than men (mOR = 1.9, 95% CI = 1.59–2.26, p<0.001). The risk of infection decreased as socio-economic status increased (mOR = 0.7, 95% CI = 0.55–0.79, p<0.001).

The consumption of river fish was significantly associated with *O*. *felineus* infection. Of the 488 enrolled participants, 431 (88.3%) reported regularly consuming river fish. Among those, 63.1% were infected *versus* 38.6% of those who did not regularly consume fish (aOR = 2.4, 95% CI = 1.52–3.95, p<0.001).

**Fig 9:**
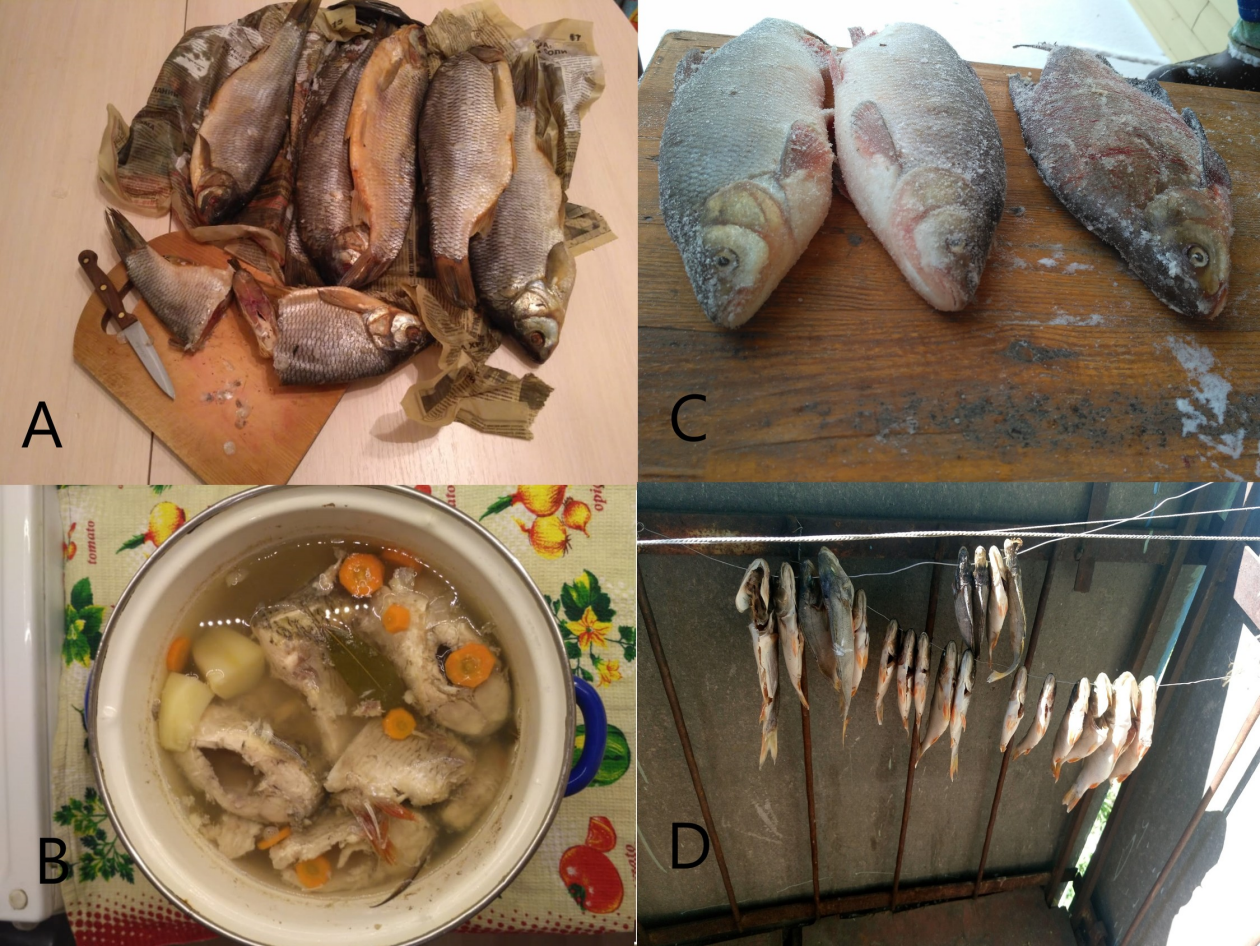
Traditional *Cyprinidae* fish in Shegarskiy district, Tomsk Oblast. A. Salty fish; B. Boiled fish (fish soup); C. Frozen fish; D. Sun-dried fish / stock fish.

Multivariate analysis showed a strong and significant increase of infection risk with the consumption of river fish dishes. For example, the risk of *O*. *felineus* infection increased with the consumption of stock fish (mOR = 3.2, 95% CI = 2.63–3.80, p<0.001), frozen fish (mOR = 1.6, 95% CI = 1.29–2.02, p<0.001), smoked fish (mOR = 1.5, 95% CI = 1.24–1.72, p<0.001) and boiled fish (mOR = 1.5, 95% CI = 1.23–1.94, p<0.001, Figs 8 and 9). The risk of *O*. *felineus* infection decreased with a lower frequency of fish consumption. Those who reportedly consumed their last fish one year ago had a significantly lower risk of *O*. *felineus* infection (mOR = 0.5, 95% CI = 0.43–0.68, p<0.001). Smoking (mOR = 2.3, 95% CI = 1.91–2.81, p<0.001) and regularly drinking alcohol (mOR = 1.9, 95% CI = 1.9–2.25, p<0.001) increased the risk of infection. Study participants who reported having a dog in the household had a significantly increased *O*. *felineus* infection prevalence (mOR = 2.1, 95% CI = 1.80–2.50, p<0.001).

### Awareness of *Opisthorchis felineus*

We analyzed the knowledge and awareness of opisthorchiasis with the question “Have you ever heard about opisthorchiasis?”. Almost all of the respondents (93.2%) and infected participants (96.6%) were aware of opisthorchiasis, and had heard about the disease before. Children aged 7–18 years had a lower awareness of *O*. *felineus* infection compared to adults 19 years and older (63.5% vs 98.6%, aOR 39.1, 95% CI 15.5–98.2, p<0.001). The majority of respondents (both infected and non-infected) indicated the consumption of undercooked river fish as the main cause of opisthorchiasis (93.0% and 83.6%, respectively). More than half of the respondents suggested other sources of infection, including drinking infected water (69.7%), swimming in infected water (60.9%), and having contact with an infected person (52.5%) or animals (77.8%). There was no significant difference in knowledge between groups of infected and non-infected individuals.

The study participants knew relatively little about the symptoms of the disease and did not understand the health consequences of the infection. Liver disease as a consequence of *O*. *felineus* infection was reported significantly more often by patients with opisthorchiasis (86.6%) compared to non-infected participants (77.2%, aOR 1,43, 95% CI 1.02–2.03, р = 0.04). Other symptoms, like stomach pain, diarrhea, skin or respiratory problems, were mentioned by less than half of the respondents (no difference between groups was observed). A possible link between *O*. *felineus* infection and cancer was indicated by 27.1% and 25.2% of infected and non-infected participants, respectively (no significant difference, corrected p = 0.63).

**Fig 10:**
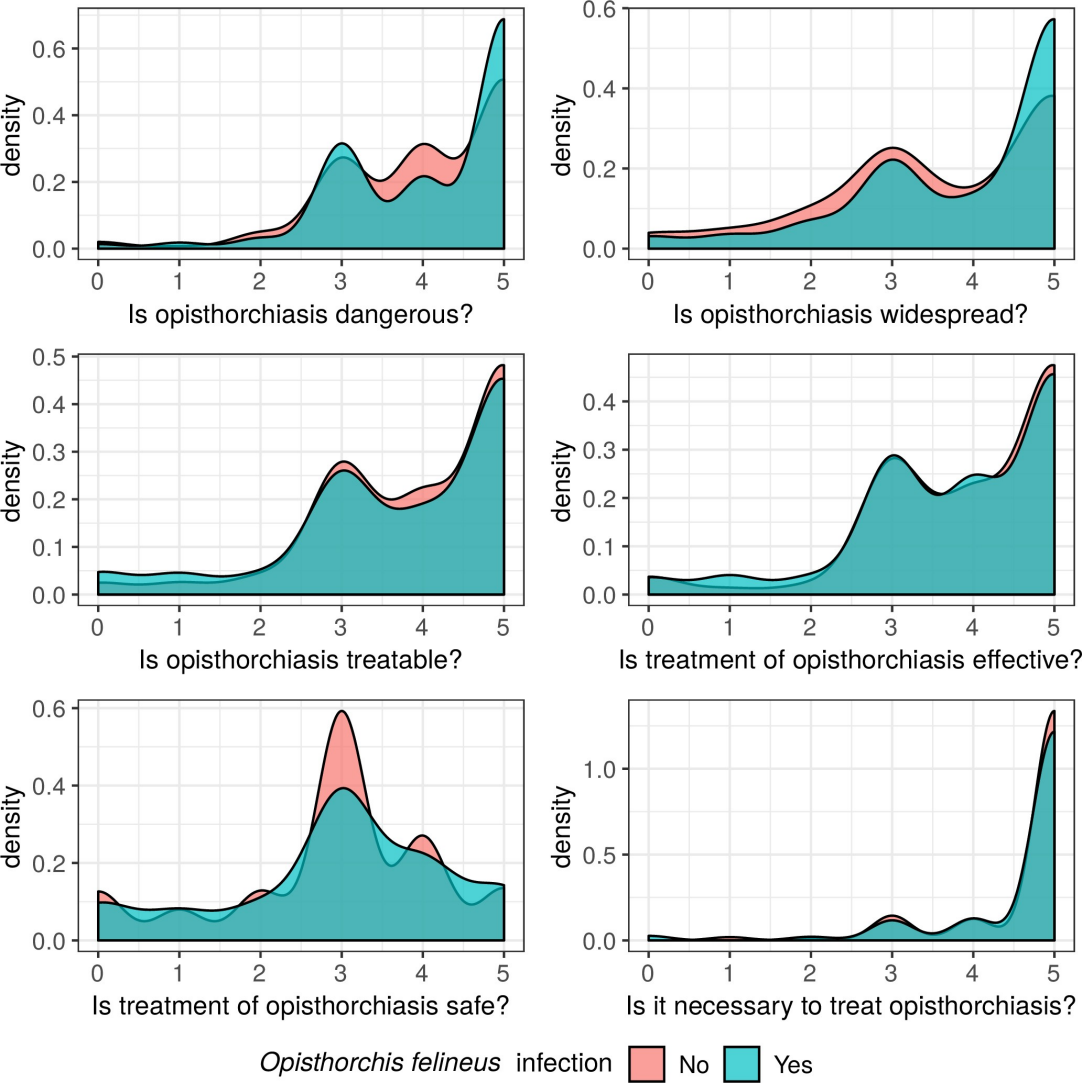
Awareness of opisthorchiasis among *O*. *felineus-*infected (green area) and non-infected (red area) study participants. A. Is opisthorchiasis dangerous for your health? (x-axis score: not dangerous at all 0-1-2-3-4-5 fatal), B. Is opisthorchiasis widespread in your setting? (x-axis score: very rare 0-1-2-3-4-5 very widespread), C. Is opisthorchiasis treatable? (x-axis score: not treatable 0-1-2-3-4-5 entirely treatable), D. Is the treatment of opisthorchiasis effective? (x-axis score: not effective 0-1-2-3-4-5 absolutely effective), E. Is the treatment of opisthorchiasis safe? (x-axis score: not safe 0-1-2-3-4-5 absolutely safe). F. Is it necessary to treat opisthorchiasis when it is diagnosed? (x-axis score: not necessary 0-1-2-3-4-5 absolutely necessary); Y-axis: density in percent (%) of total.

To analyze the perception of opisthorchiasis by the population of an endemic region, different analogue scales from 0–5 points were used (scale ranged from “do not agree” or “0”, to “completely agree” or “5”; Fig 10). More than half of the study participants perceived opisthorchiasis as a dangerous and widespread disease. Most of the respondents answered in the affirmative to the question, “Is opisthorchiasis treatable?”. However, the safety of treatment with the current drug (praziquantel) was perceived as being moderate.

Among those who had heard about opisthorchiasis, 95.6% were willing to undergo treatment in case of a diagnosed *O*. *felineus* infection. Furthermore, 98.4% were ready to follow the doctor’s recommendation and only 4.6% considered asking relatives and consulting the internet before treatment. Among the respondents, 87.0% indicated that they would follow the recommendations to take praziquantel, 53.9% thought it acceptable to use dietary supplements for treatment, and 2.6% pointed to some other form of treatment, like folk remedies and herbal tinctures.

Respondents suggested that opisthorchiasis may be effectively prevented by cooking fish thoroughly (76.1%) and avoiding river fish consumption (52%). There was no difference between groups with different infection status. Using separate cooking tools (60.4%), washing hands (75.2%) and drinking pre-boiled water (63.0%) were also reported as effective preventive measures against infection. At the same time, 20.9% of respondents indicated that it is impossible to prevent infection in an endemic region (24.4% among infected vs 15.0% among non-infected individuals, aOR 1.7, 95% CI 1.09–2.63, р = 0.02).

### Clinical examination and abdominal ultrasonography

**Fig 11:**
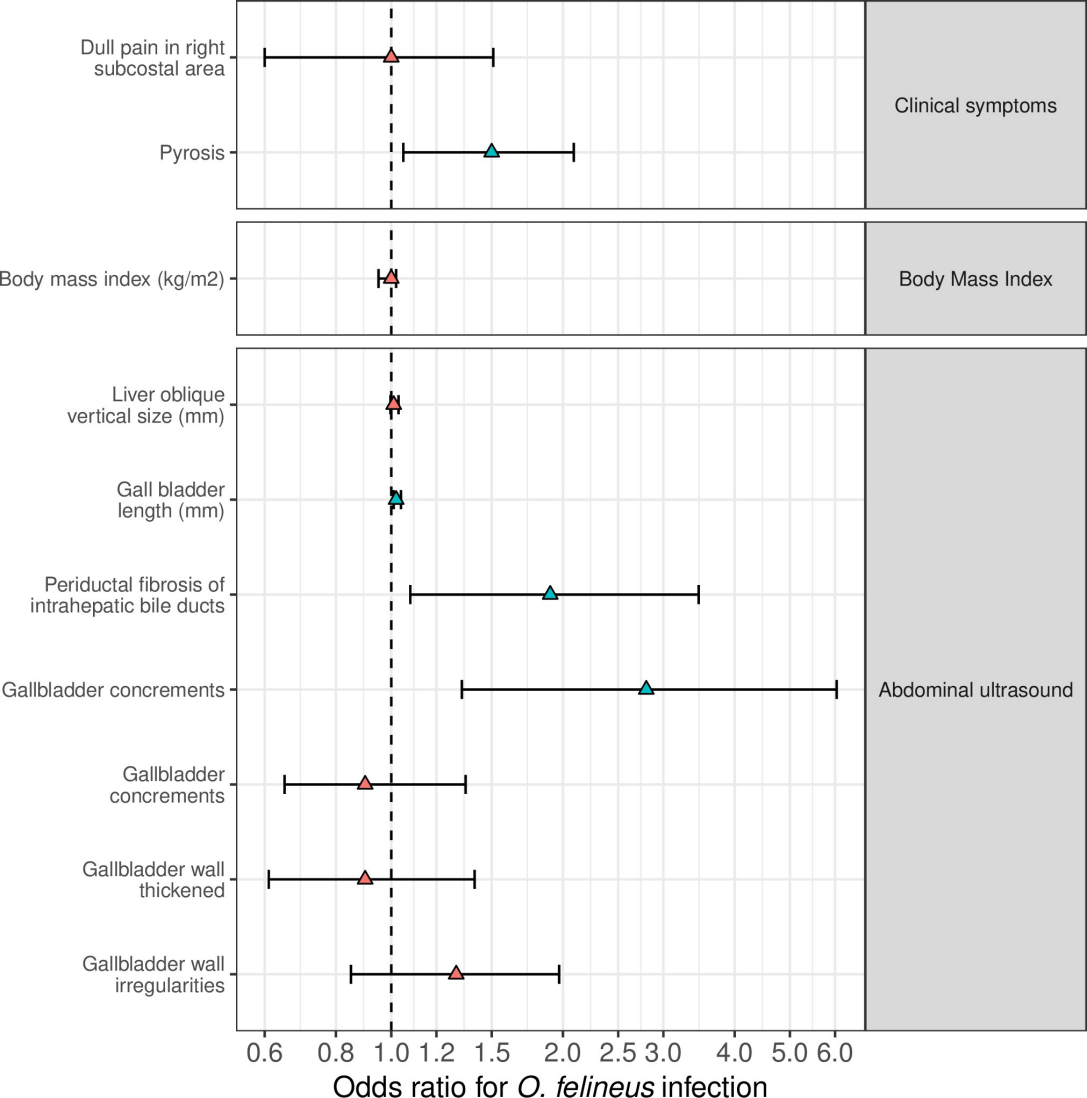
Morbidity associated with *Opisthorchis felineus* infection. Odds ratio (OR, triangles) and 95% confidence intervals of OR (95% CI, bars) in logarithmic scale (base 5); green and red triangles for statistically significant (p ≤ 0.05) and not significant (p > 0.05) OR.

Among *O*. *felineus* infected people, the most common complaint was a dull pain in the right subcostal area. There was no statistically significant difference with non-infected participants (Fig 11). Pyrosis was observed in 55.9% of the infected individuals and was associated with opisthorchiasis (mOR 1.5, 95% CI 1.05–2.09, p = 0.03). Headache, fatigue, and vertigo were reported in both *O*. *felineus*-positive and -negative study participants and were not associated with any statistical significance.

The findings of the abdominal ultrasonography examination (related to the liver parenchyma, the gallbladder and the bile ducts) are presented in Fig 11 (details in S4 Table). Several pathological features of the gallbladder were observed more frequently in *O*. *felineus*-infected *versus* non-infected participants. For example, gallbladder wall irregularities (42.3% *vs* 25.6%), wall thickening (74.7% *vs* 56.9%) and presence of a halo in the gallbladder (68.5% *vs* 55.0%). However, the calculated risk increase was only significant in the univariate analysis and absent in the multivariate analysis. But, the presence of a gallbladder stones was strongly associated with *O*. *felineus* infection; this abnormality was present in 16.9% of infected study participants and only in 4.4% of the uninfected study participants (mOR = 2.8, 95% CI = 1.33–6.04, p = 0.007).

Increased echogenicity of liver tissue was found predominantly in *O*. *felineus*-infected patients (76.2%) compared to non-infected participants (52.1%) and was significantly associated in the univariate analysis (aOR = 2.9, 95% CI = 2.00–4.15, p<0.001).

An intrahepatic bile duct dilatation was diagnosed in 20 (4.6%) study participants (6% infected; 3% non-infected). The enlargement was not associated with the *Opisthorchis* infection status of the study participants (OR = 1.8, 95% CI = 0.66–4.80, p = 0.259). Periductal fibrosis of intrahepatic bile ducts was significantly and strongly associated with an *O*. *felineus* infection: 17.7% and 74.3% of the infected study participants had a grade 2 and grade 1 periductal fibrosis in intrahepatic bile ducts, respectively. Of the non-infected study participants, only 10.5% and 55.0% yielded the same finding. Only 7.9% of the infected patients showed no intrahepatic periductal fibrosis compared to 34.5% in the uninfected group. Thickened gallbladder walls were found among 74.7% of the patients with *O*. *felineus* infection, and among 56.9% of participants without infection. The difference was not statistically significant.

## Discussion

*O*. *felineus* infection causes a chronic inflammatory disease of the bile ducts. This trematode parasite is widely spread throughout the Russian Federation, with the highest prevalence rates reported in the middle and lower streams of the Ob and Irtysh rivers (Western Siberia) [2]. Analysis of the official medical data indicates that *O*. *felineus* infection in some regions of the Russian Federation may reach as many as 600 new infected patients per 100 000 per year [3,5]. However, information on the extent and importance of *O*. *felineus* infection prevalence, risk factors and associated morbidity in rural communities is hardly available. There is a need for greater understanding of the endemic setting in terms of the current infection and morbidity burdens and associated risk factors in order to develop an adequate control program.

We conducted a cross-sectional study in the rural Shegarskiy district, Tomsk Oblast, Western Siberia, to estimate the prevalence, intensity, risk factors, and clinical significance of *O*. *felineus* infection in this endemic community. A total of 600 randomly selected individuals, aged seven years and older, were enrolled from nine villages in the district, 488 (81.3%) persons actively participated at the study and of them 436 (72.7%) had US examination. Several assessments were performed on each study participant to assess infection and morbidity and risk factor profiles.

In our study, we found a high prevalence of *O*. *felineus* infection (60.2%). The vast majority of the infected individuals had low-intensity infections (70.4%), but 3.1% of them had high-intensity infections. *O*. *felineus* infection was significantly associated with increasing age and being female, and negatively associated with socio-economic status. Furthermore, the consumption of river fish, particularly undercooked fish; fishing activities; smoking; consumption of alcohol; and presence of dogs in the household were significantly associated with an increased risk of infection. Usually people feed dogs by undercooked river fish, and this could be resulted on the contamination. Dogs are appropriate definitive hosts of *O*. *felineus* and might play a role in the transmission of *O*. *felineus* in Siberia [1]. Despite a high awareness of opisthorchiasis (93.2%), our work highlights the public’s insufficient knowledge related to infection transmission and prevention. Children and adolescents had lower levels of awareness of infection compared to adults. Our study also assessed the impact that *O*. *felineus* infection has on human health. The abdominal ultrasonography results demonstrated a strong association between *O*. *felineus* infection and hepatobiliary pathology, such as gallbladder stones, and intrahepatic periductal fibrosis. In a considerable number of participants, an intrahepatic bile duct dilation was observed.

### Prevalence of *Opisthorchis felineus* infection

This is the first community-based study assessing the epidemiology of *O*. *felineus* infection and morbidity in the endemic region of Western Siberia, Russian Federation, in the 21st century. According to a recently published Russian-language literature review, a number of studies focused on the occurrence of infection in Western Siberia some 20–30 years ago. They showed that *O*. *felineus* prevalence exceeded 50% among adults in some Siberian regions, such as Tomsk Oblast, Tyumen Oblast, and Yamal-Nenets and Khanty-Mansiysk autonomous Okrugs [10]. Our results show that present-day *O*. *felineus* infection prevalence in Shegarskiy district is still (at least) as high as indicated in previous reports (over 60%). Hence, there is a need to revise the current control measures aiming to prevent, diagnose and treat the infection.

Our study documents ongoing transmission of *O*. *felineus* in rural Western Siberia, leading to infected humans, wildlife and fresh water fish. By way of these hosts, the helminth is transported to other areas with suitable environmental features. Indeed, analysis of official epidemiological data suggests that, over the two last decades, *O*. *felineus* infection has spread to new, previously non-endemic regions [3]. Given high international trade and travel level (in rural Western Siberia), the export of *O*. *felineus* infection to other areas in Europe, for example, is likely to happen. Recently published papers described the cases of opisthorchiasis in citizens of European Russia after short staying in endemic Siberian regions [30,31]. Our findings underscore the public health importance of *O*. *felineus* infection in rural Western Siberian settings.

We observed that the prevalence and intensity of infection increased until the age of about 40 years. We also observed that infection prevalence was highly and positively associated with infection intensity. The diagnosis and treatment of *O*. *felineus* infection is rather rare in Shegarskiy district. As the population is continuously exposed to new infections, the infection accumulates over time, resulting in high infection prevalence and intensities in older age groups. We have observed this previously for *O*. *felineus* [10]. This observation is also common in rural settings in Southeast Asia where *O*. *viverrini* is endemic [32,33].

### Risk factors for *Opisthorchis felineus* infections

The main risk factor for an *O*. *felineus* infection is the consumption of freshwater fish from the *Cyprinidae* family, such as Ide (*Leuciscus idus*), Common dace (*Leuciscus leuciscus*), Bream (*Abramis* spp.), Crucian (*Carassius carassius*), Carp (*Cyprinus* spp.), and others. The majority of infected participants ate undercooked fish, be it smoked, stocked / sun-dried, frozen, or raw. They also reported consuming fried and boiled fish. This dietary tradition is connected to other behavioral risk factors, such as fishing activities, smoking, and alcohol consumption. *O*. *felineus* transmission is driven by the importance of locally caught river fish for nutrition, leisure, and community building, as a part of the local “life by the river” [22]. Results of studies in Thailand and Italy showed that eating habits, together with insufficient knowledge of infection in the community are the key risk factors for acquiring an *Opisthorchis* infection [33–35].

In contrast to studies in Southeast Asia, we did not find an association between availability of adequate sanitation facilities in the household and fluke infection. In all Siberian households, toilets and latrines are present and people are in the habit of using them indoors. Open (outdoor) defecation outside of the household is rare, unlike in *O*. *viverrini* endemic settings in Southeast Asia. In the Western Siberian setting and in Europe, wildlife is mainly responsible for the transmission of the parasite to fish; *O*. *felineus* infection in humans is zoonotic in origin unlike *O*. *viverrini* and *C*. *sinensis* which are more anthroponotic [1,6].

According to our study, women are at a higher risk of being infected than men. This is most likely due to their involvement in preparing and cooking fish. We found that infection was negatively associated with the socio-economic status of the population. This finding corresponds to the results of epidemiological studies of other liver-fluke-endemic settings, such as Southeast Asia, and of other NTDs in general [32,36].

### Awareness of *Opisthorchis felineus* infection

Our study demonstrates a high level of awareness of opisthorchiasis among adult rural inhabitants. Thus, future awareness programs and campaigns should primarily target children and adolescents.

Our study revealed some misconceptions about the transmission of *O*. *felineus* infection. For example, beliefs that one can become directly infected with *O*. *felineus* by contact with "dirty hands", or with infected people and animals. Among the methods for preventing opisthorchiasis, participants indicated hand washing, and pre-boiling drinking water. In addition, almost one in five respondents (18.7%) thought that preventing fluke infection in an endemic region was not possible. These observations suggest that any future control strategies must include community-focused information, education, and communication campaigns to address these misconceptions around *O*. *felineus* transmission and prevention.

### Morbidity associated with *Opisthorchis felineus* infection

In our study, infected individuals suffered from nonspecific abdominal (upper right quadrant) pain. Half of the patients experienced heartburn, which was most probably gastroesophageal reflux associated with *O*. *felineus* infection. Other common complaints included general malaise, weakness, headache, itching, and skin rash, but these symptoms were not strongly specific. Previous research showed that *O*. *felineus* infection may be asymptomatic, yet chronic infections may result in severe complications. The clinical consequences depend on the intensity and duration of cumulative infestations [37,38]. We could not identify clinical symptoms associated with *O*. *felineus* infection. Many factors could account for this finding; the non-specificity of the symptoms and reporting bias would be among them.

During abdominal ultrasound examinations, we observed a clear association between *O*. *felineus* infection and pathology of the liver bile ducts and gallbladder. For instance, infected individuals had a high prevalence of gallbladder stones. An increased echogenicity of liver parenchyma was another sign of *O*. *felineus* infection. Abnormality of liver bile ducts (e.g. periductal fibrosis) was strongly associated with an *O*. *felineus* infection. In our study cohort, we did not identify patients with a liver mass or suspected cholangiocarcinoma but in 20 participants an enlarged intrahepatic bile duct dilation was observed.

A long-lasting fluke infection may cause injury to the bile ducts, while their metabolic products irritate the biliary epithelial cells. Long-lasting inflammation may lead to cell desquamation, hyperplasia, eventual periductal fibrosis and bile duct dilatation [37,39]. Chronic massive *O*. *felineus* infection accompanied by sclerotic changes in the walls of the ducts leads to the development of strictures and worsening of bile passage, which can lead to mechanical cholestasis requiring surgical treatment. According to the International Agency for Research on Cancer, liver flukes from the Opisthorchiidae family are classified as cancerogenic agents [40]. There are also well-documented studies showing the association between *O*. *viverrini* infection and fatal bile duct cancer–cholangiocarcinoma in Southeast Asia [38,41].

The cancerogenic potential of the *O*. *felineus* fluke has been described in experimental studies and clinical reports [37,39,42]. A review of official medical statistics presented an association between *O*. *felineus* infection and liver or bile duct cancer incidence rates in endemic areas [3]. To assess the morbidity connected to *O*. *felineus* infection, we used abdominal ultrasonography of the liver parenchyma and bile ducts. Reports from epidemiological studies in Thailand documented the importance of periductal fibrosis and bile duct dilatation as ultrasonography markers when screening a population at risk of cholangiocarcinoma [25,38].

## Study limitations

We restricted our study to rural areas of Western Siberia and selected the Shegarskiy district, specifically. Therefore, we were not able to estimate the infection burden of urban centers, such as Tomsk.

We detected eggs of *O*. *felineus* in stool samples by microscopy by use of PARASEP, an application of formalin-ethyl acetate concentration technique (DiaSys Ltd, UK). Previous studies demonstrated that PARASEP has a higher sensitivity than Kato-Katz and McMaster techniques for detecting *Schistosoma mansoni*, *Ascaris lumbricoides*, and *Hymoenolepis nana* [43,44]. However, in Thailand the technique showed a low sensitivity for the detection of light infections *O*. *viverrini* which increased higher infection intensities (at ≥ 50 EPG) [45]. We restricted our diagnostic approach to egg detection. It has previously been reported that egg shedding of *O*. *felineus* adult may be interrupted. In our study, we collected and examined two stool samples per person, which has been shown to increase the sensitivity for *Opisthorchis* egg detection [46]. Where possible, e.g. in central hospital settings, molecular techniques might be a more sensitive and appropriate diagnostic approach. All the stool samples were investigated by one experienced and specially trained medical parasitologist, according to protocol and PARASEP manufacturer’s instruction sheet. Eggs of *O*. *felineus* are morphologically similar to eggs of *Metorchis spp*. a close relative of *O*. *felineus* [47]. However, latter trematodes are only occasionally diagnosed in the Russian Federation and hence might have only marginally contributed to a confusion of the results.

## Conclusion

Our cross-sectional study documents the high *O*. *felineus* liver fluke infection and hepatobiliary morbidity burden in a rural area of Western Siberia, Russian Federation. Social and dietary factors play a determinant role in the transmission of *O*. *felineus* in endemic rural regions. Abdominal US assessments document severe hepatobiliary morbidity. Therefore, *O*. *felineus* infection and the associated morbidity is most probably much more frequent than currently thought. Our results will be used to develop a multidisciplinary community-based control program against *O*. *felineus* infection in endemic regions.

## Supporting information

S1 Checklist STROBE checklist. (DOC)

S1 Table *Opisthorchis felineus* infection rates overall and stratified by sub-groups (N = 488). (DOCX)

S2 Table *Opisthorchis felineus* infection intensity overall and stratified by different groups. (DOCX)

S3 Table Risk factors of *Opisthorchis felineus* infection (results of the univariable and multivariable analysis). (DOCX)

S4 Table Morbidity associated with *Opisthorchis felineus* infection (results of the univariable and multivariable analysis). (DOCX)
