# Supplementary material for: Opisthorchis felineus infection, risks, and morbidity in rural Western Siberia, Russian Federation
Source: PLoS Negl Trop Dis. 2020 Jun 29;14(6):e0008421. doi: 10.1371/journal.pntd.0008421 (PMC7351239; doi:10.1371/journal.pntd.0008421)
Supplement: S1 Checklist — (DOC) [file pntd.0008421.s001.doc]

**S1 Checklist: STROBE Checklist**

|  | No | Checklist |
| --- | --- | --- |
| **Title and abstract** | 1 | (*a*) We used term of the study’s design in the title and abstract |
|  |  | (*b*) We provided in the abstract the summary of what was done and what was found |
| Introduction | | |
| Background/rationale | 2 | We explained the scientific background and rationale of the investigation |
| Objectives | 3 | We included the aim of the study |
| Materials and methods | | |
| Study design | 4 | “Materials and methods”, “Study population and design”, paragraph 1 |
| Setting | 5 | “Materials and methods”, “Study setting”, paragraph 1-4 |
| Participants | 6 | “Materials and methods”, “Study population and design”, paragraph 1-2 |
| Variables | 7 | “Materials and methods” – “Field and laboratory procedures”; “Data management and statistical analysis”, paragraph 4 |
| Data sources/ measurement | 8* | “Materials and methods” – “Field and laboratory procedures” |
| Bias | 9 | “Study population and design”, “Field and laboratory procedures” |
| Study size | 10 | “Materials and methods”, “Study population and design”, paragraph 1-2, Fig. 2 |
| Quantitative variables | 11 | “Data management and statistical analysis”, paragraph 4 |
| Statistical methods | 12 | “Materials and methods”, “Data management and statistical analysis”, paragraph 1-5 |
| Results | | |
| Participants | 13* | (a) Report numbers of individuals at each stage of study – “Results”, “Study population”, paragraph 1-2, Fig. 2 |
|  |  | (b) Give reasons for non-participation at each stage – “Results”, “Study population”, paragraph 1-2, Fig. 2 |
|  |  | (c) flow diagram - Fig. 2 |
| Descriptive data | 14* | (a) characteristics of study participants (eg demographic, clinical, social) and information on exposures and potential confounders – “Results”, “Study population”, paragraph 1-2, Table 1 |
|  |  | (b) Indicate number of participants with missing data for each variable of interest – Tables S1-4 |
| Main results | 15 | (*a*) Unadjusted estimates and confounder-adjusted estimates and their precision (eg, 95% confidence interval) were given in “Prevalence of *Opisthorchis felineus* infection”, “Risk factors for *Opisthorchis felineus* infection”, “Awareness of *Opisthorchis felineus*”, “Clinical examination and abdominal ultrasonography”, and tables S3-S4. |
|  |  | (*b*) “Data management and statistical analysis”, paragraphs 1 and 3 |
|  |  | (*c*) Not relevant |
| Other analyses | 17 | none |
| Discussion | | |
| Key results | 18 | “Discussion”, paragraph 1-2, |
| Limitations | 19 | “Discussion”, “Study limitations” |
| Interpretation | 20 | “Discussion”, “Prevalence of Opisthorchis felineus infection”, “Risk factors for Opisthorchis felineus infections”, “Awareness of Opisthorchis felineus infection”, “Morbidity associated with Opisthorchis felineus infection” |
| Generalisability | 21 | “Risk factors for *O. felineus* infection”, “Awareness of *O. felineus* infection”, “Morbidity associated with *O. felineus* infection” |
| Other information | | |
| Funding | 22 | “Funding” |
