# Supplementary material for: Opisthorchis felineus infection, risks, and morbidity in rural Western Siberia, Russian Federation
Source: PLoS Negl Trop Dis. 2020 Jun 29;14(6):e0008421. doi: 10.1371/journal.pntd.0008421 (PMC7351239; doi:10.1371/journal.pntd.0008421)
Supplement: S1 Table — (DOCX) [file pntd.0008421.s002.docx]

**Table S1. *Opisthorchis felineus* infection rates overall and stratified by sub-groups (N = 488)**

|  |  | Total | % | Male  (n=147) | % | Female (n = 341) | % |
| --- | --- | --- | --- | --- | --- | --- | --- |
| *O.* ***felineus*** positive  (95% CI) a | Overall | 294 | 60.2  (57.3-63.1)^a^ | 82 | 55.8  (51.0-60.5)^a^ | 212 | 62.2  (59.1-65.2) ^a^ |
| Age | 7-11 | 4 | 10.0 | 1 | 5.6 | 3 | 13.6 |
|  | 12-18 | 8 | 23.5 | 3 | 21.4 | 5 | 25.0 |
|  | 19-39 | 66 | 71.7 | 18 | 78.3 | 48 | 69.6 |
|  | 40-59 | 121 | 67.6 | 36 | 72.0 | 85 | 65.9 |
|  | >60 | 95 | 66.4 | 24 | 57.1 | 71 | 70.3 |
| Village | Batkat | 31 | 62.0 | 6 | 40.0 | 25 | 71.4 |
|  | Kargala | 16 | 76.2 | 5 | 83.3 | 11 | 73.3 |
|  | Malobragino | 16 | 80.0 | 3 | 100.0 | 13 | 76.5 |
|  | Melnikovo | 96 | 52.2 | 23 | 46.0 | 73 | 54.5 |
|  | Monostyrka | 15 | 71.4 | 5 | 55.6 | 10 | 83.3 |
|  | Novoiljinka | 18 | 90.0 | 4 | 80.0 | 14 | 93.3 |
|  | Pobeda | 72 | 60.0 | 30 | 66.7 | 42 | 56.0 |
|  | Voronovka | 16 | 47.1 | 2 | 25.0 | 14 | 53.8 |
|  | Vosnesenka | 14 | 77.8 | 4 | 66.7 | 10 | 83.3 |
| Education | Incomplete secondary education | 35 | 49.3 | 8 | 33.3 | 27 | 57.5 |
|  | Secondary education | 85 | 73.3 | 20 | 66.6 | 65 | 75.6 |
|  | Technical education | 97 | 70.3 | 29 | 74.4 | 68 | 68.7 |
|  | High education | 60 | 55.1 | 16 | 59.3 | 44 | 53.7 |
|  | Unknown | 17 | 31.5 | 9 | 33.3 | 8 | 29.6 |
| Employment at moment | Yes | 142 | 67.9 | 41 | 73.2 | 101 | 66.0 |
|  | No | 152 | 54.5 | 41 | 45.1 | 111 | 59.0 |
| Socioeconomic status | Lowest | 81 | 77.9 | 22 | 75.9 | 59 | 78.7 |
|  | Low | 65 | 65.0 | 11 | 52.4 | 54 | 68.4 |
|  | Moderate | 74 | 67.3 | 25 | 64.1 | 49 | 69.0 |
|  | High | 63 | 62.4 | 20 | 76.9 | 43 | 57.3 |

______________

^a^ - Number in brackets indicates the 95% Confidence Interval (95% CI)
