# Supplementary material for: Opisthorchis felineus infection, risks, and morbidity in rural Western Siberia, Russian Federation
Source: PLoS Negl Trop Dis. 2020 Jun 29;14(6):e0008421. doi: 10.1371/journal.pntd.0008421 (PMC7351239; doi:10.1371/journal.pntd.0008421)
Supplement: S2 Table — (DOCX) [file pntd.0008421.s003.docx]

**Table S2. *Opisthorchis felineus* infection intensity overall and stratified by different groups**

| **Groups** | | **Total**  **(geom. mean)** | **Total**  **(Range)** | **Male**  **(geom. mean)** | **Male**  **(Range)** | **Female**  **(geom. mean)** | **Female**  **(Range)** |
| --- | --- | --- | --- | --- | --- | --- | --- |
| Overall infection intensity | | 283 | 2-43200 | 181 | 4-25200 | 337 | 2-43200 |
| Age | 7-11 | 59 | 12-444 | 36 | 36 | 69 | 12-444 |
|  | 12-18 | 27 | 6-180 | 15 | 6-24 | 38 | 6-180 |
|  | 19-39 | 184 | 2-6800 | 99 | 4-5700 | 231 | 2-6800 |
|  | 40-59 | 333 | 2-43200 | 207 | 6-25200 | 407 | 2-43200 |
|  | 60 and above | 325 | 6-32940 | 230 | 10-23160 | 366 | 6-32940 |
| Village | Batkat | 395 | 6-32940 | 300 | 10-12348 | 422 | 6-32940 |
|  | Kargala | 348 | 8-5112 | 171 | 8-3276 | 480 | 48-5112 |
|  | Malobragino | 1201 | 8-43200 | 1895 | 90-25200 | 1081 | 8-43200 |
|  | Melnikovo | 163 | 2-36000 | 79 | 6-1272 | 204 | 2-36000 |
|  | Monostyrka | 308 | 24-4740 | 320 | 24-1470 | 302 | 24-4740 |
|  | Novoiljinka | 1157 | 20-15900 | 2833 | 378-15900 | 896 | 20-12600 |
|  | Pobeda | 148 | 4-7600 | 92 | 4-7600 | 208 | 6-6400 |
|  | Voronovka | 467 | 6-6528 | 108 | 36-324 | 575 | 6-6528 |
|  | Vosnesenka | 482 | 6-23160 | 1172 | 156-23160 | 338 | 6-8640 |
| Education | Incomplete secondary education | 354 | 8-43200 | 115 | 8-1880 | 494 | 16-43200 |
|  | Secondary education | 338 | 4-23160 | 231 | 4-23160 | 380 | 6-8640 |
|  | Technical education | 333 | 6-36000 | 208 | 6-25200 | 407 | 6-36000 |
|  | High education | 122 | 2-8000 | 97 | 12-1512 | 133 | 2-8000 |
|  | Unknown | 164 | 6-7320 | 110 | 6-7320 | 259 | 6-4680 |
| Employment at moment | Yes | 282 | 2-43200 | 172 | 4-25200 | 338 | 2-43200 |
|  | No | 246 | 2-36000 | 152 | 8-12348 | 298 | 2-36000 |
| Socioeconomic status | lowest | 445 | 4-43200 | 287 | 4-23160 | 525 | 6-43200 |
|  | low | 410 | 6-15900 | 223 | 10-15900 | 464 | 6-8490 |
|  | moderate | 214 | 2-36000 | 203 | 6-25200 | 219 | 2-36000 |
|  | high | 151 | 2-32940 | 83 | 8-1512 | 199 | 2-32940 |
