# Supplementary material for: Opisthorchis felineus infection, risks, and morbidity in rural Western Siberia, Russian Federation
Source: PLoS Negl Trop Dis. 2020 Jun 29;14(6):e0008421. doi: 10.1371/journal.pntd.0008421 (PMC7351239; doi:10.1371/journal.pntd.0008421)
Supplement: S3 Table — (DOCX) [file pntd.0008421.s004.docx]

**Table S3. Risk factors of *Opisthorchis felineus* infection (results of the univariable and multivariable analysis)**

| **Risk factors** | | | | **O.f.^^[[1]](#endnote-1)^^ positive. n** | **%** | **Univariate** | | | | **Multivariate** | | |
| --- | --- | --- | --- | --- | --- | --- | --- | --- | --- | --- | --- | --- |
|  |  |  |  |  |  | **aOR** | **95%CI** | **P-value** | **FDR-corrected p-value** | **mOR** | **95%CI** | **P-value** |
| Age, n=488 | |  | |  |  |  | Added as covariate | |  | 1.03 | 1.02-1.04 | <0.001 |
| Gender, n=488 | | Male  Female | | 82 | 55.8 |  |  | |  |  |  |  |
|  |  |  |  | 212 | 62.2 |  | Added as covariate | |  | 1.9 | 1.59-2.26 | <0.001 |
| Socioeconomic status  n=415^2^ | | lowest  low  moderate  high | | 81/104 | 77.9 |  |  | |  |  |  |  |
|  |  |  |  | 65/100 | 65.0 |  |  | |  |  |  |  |
|  |  |  |  | 74/110 | 67.3 |  |  | |  |  |  |  |
|  |  |  |  | 63/101 | 62.4 | 0.5 | 0.37-0.80 0.002 | | 0.015 | 0.7 | 0.55-0.79 | <0.001 |
| **River fish consumption** | |  | |  |  |  |  |  |  |  |  |  |
| River fish, n=488 | | | no | 22/57 | 38.6 | 1 |  |  |  |  |  |  |
|  | | yes | | 272/431 | 63.1 | 2.4 | 1.52-3.95 | <0.001 | 0.003 | square root of VIF > 2, excluded | | |
| Fried/boiled fish, n=431^3^ | | no | | 6/15 | 40.0 | 1 |  |  |  |  |  |  |
|  | | yes | | 266/461 | 63.9 | 1.9 | 1.22-3.05 | 0.005 | 0.032 | 1.5 | 1.23-1.94 | <0.001 |
| Smoked fish, n=431^3^ | | no | | 57/119 | 47.9 | 1 |  |  |  |  |  |  |
|  | | yes | | 215/312 | 68.9 | 2.8 | 1.79-4.36 | <0.001 | <0.001 | 1.5 | 1.24-1.72 | <0.001 |
| Stock fish, n=431^3^ | | no | | 102/202 | 50.5 | 1 |  |  |  |  |  |  |
|  | | yes | | 170/229 | 74.2 | 3.3 | 2.26-4.84 | <0.001 | <0.001 | 3.2 | 2.63-3.80 | <0.001 |
| Salty fish, n=431^3^ | | no | | 117/211 | 55.5 | 1 |  |  |  |  |  |  |
|  | | yes | | 155/220 | 70.5 | 2.0 | 1.40-2.89 | <0.001 | 0.002 | 0.6 | 0.50-0.71 | <0.001 |
| Slightly salty fish, n=431^3^ | | | no | 168/290 | 57.9 | 1 |  |  |  |  |  |  |
|  | | | yes | 104/141 | 73.8 | 2.4 | 1.53-3.70 | <0.001 | 0.002 | 1.2 | 0.98-1.46 | 0.084 |
| Frozen fish, n=431^3^ | no | | | 171/300 | 57.0 | 1 |  |  |  |  |  |  |
|  | yes | | | 101/131 | 77.1 | 2.8 | 1.89-4.29 | <0.001 | <0.001 | 1.6 | 1.29-2.02 | <0.001 |
| Raw fish, n=431^3^ | no | | | 194/329 | 59.0 | 1 |  |  |  |  |  |  |
|  | yes | | | 78/102 | 76.5 | 2.5 | 1.65-3.65 | <0.001 | <0.001 | 1.4 | 1.05-1.84 | 0.022 |
| Last time of river | two days ago | | | 63/87 | 72.4 |  |  |  |  |  |  |  |
| fish consumption, | last week | | | 73/103 | 70.9 |  |  |  |  |  |  |  |
| n=431^3^ | 2-3 weeks ago | | | 26/50 | 52.0 |  |  |  |  |  |  |  |
|  | 1 month ago | | | 40/64 | 62.5 |  |  |  |  |  |  |  |
|  | more than 1 month | | | 59/105 | 56.2 |  |  |  |  |  |  |  |
|  | more than 1 year | | | 11/22 | 50.0 | 0.4 | 0.24-0.64 | <0.001 | 0.002 | 0.5 | 0.43-0.68 | <0.001 |
| **Other risk factors** |  | | |  |  |  |  |  |  |  |  |  |
| Fisherman in the | no | | | 104/188 | 55.3 | 1 |  |  |  |  |  |  |
| house, n=462^4^ | yes | | | 178/274 | 65.0 | 1.9 | 1.31-2.63 | 0.001 | 0.005 | 0.9 | 0.79-1.13 | 0.516 |
| Fishing (personally) | no | | | 158/268 | 59.0 | 1 |  |  |  |  |  |  |
| n=431^3^ | yes | | | 113/163 | 69.3 | 2.0 | 1.31-2.99 | 0.001 | 0.010 | 1.2 | 1.03-1.43 | 0.019 |
| Passive smoking | no | | | 129/236 | 54.7 | 1 |  |  |  |  |  |  |
| n=488 | yes | | | 165/252 | 65.5 | 1.6 | 1.23-2.20 | 0.001 | 0.007 | 0.9 | 0.81-1.08 | 0.370 |
| Smoking currently | no | | | 222/399 | 55.6 | 1 |  |  |  |  |  |  |
| n=488 | yes | | | 72/89 | 80.9 | 3.9 | 2.39-6.36 | <0.001 | <0.001 | 2.3 | 1.91-2.81 | <0.001 |
| Drinking alcohol | no | | | 80/189 | 42.3 | 1 |  |  |  |  |  |  |
| n=488 | yes | | | 214/299 | 71.6 | 3.0 | 2.06-4.24 | <0.001 | <0.001 | 1.9 | 1.58-2.25 | <0.001 |
| Dogs in the house | no | | | 176/267 | 65.9 | 1 |  |  |  |  |  |  |
| n=488 | yes | | | 118/221 | 53.4 | 2.2 | 1.43-3.45 | <0.001 | 0.004 | 2.1 | 1.80-2.50 | <0.001 |

_________________________

- O.f. - *Opisthorchis felineus*

^2^ – Children under 18 years were excluded, n=73

^3^ – in subsample of persons who answered “Yes” to question “Did you ever eat river fish?”, n=431

^4^ – in subsample of persons who answered “Yes” to question “Does anyone in your house consume river fish?”, n=462

aOR – Odds ratio adjusted for age and sex

mOR – Odds ratio from the multivariable analysis

VIF – Variance inflation factor

1. [↑](#endnote-ref-1)
