# Supplementary material for: Opisthorchis felineus infection, risks, and morbidity in rural Western Siberia, Russian Federation
Source: PLoS Negl Trop Dis. 2020 Jun 29;14(6):e0008421. doi: 10.1371/journal.pntd.0008421 (PMC7351239; doi:10.1371/journal.pntd.0008421)
Supplement: S4 Table — (DOCX) [file pntd.0008421.s005.docx]

**Table S4. Morbidity associated with *Opisthorchis felineus* infection (results of the univariable and multivariable analysis)**

|  | | **Total, n** | **%** | ***O. f.^^[[1]](#endnote-1)^^* positive, n** | **%** | ***O. f.* negative, n** | **%** | **Univariate** | | | | **Multivariate** | | |
| --- | --- | --- | --- | --- | --- | --- | --- | --- | --- | --- | --- | --- | --- | --- |
|  |  |  |  |  |  |  |  | **OR** | **CI 95%** | **P-value** | **FDR-corrected p-value** | **OR** | **CI 95%** | **P-value** |
| **Clinical symptoms** |  |  |  |  |  |  |  |  |  |  |  |  |  |  |
| Pyrosis, n=436 | no | 216 | 49.5 | 117 | 44.2 | 99 | 57.9 | 1 |  |  |  |  |  |  |
|  | yes | 220 | 50.5 | 148 | 55.9 | 72 | 42.1 | 1.7 | 1.22-2.26 | 0.001 | 0.016 | 1.5 | 1.05-2.09 | 0.026 |
| Dull pain in right subcostal area, n=200 | no | 118 | 59.0 | 62 | 52.1 | 56 | 69.1 | 1 |  |  |  |  |  |  |
|  | yes | 82 | 41.0 | 57 | 47.9 | 25 | 30.9 | 2.1 | 1.33-3.26 | 0.001 | 0.016 | 1.0 | 0.60-1.51 |  |
| **Abdominal ultrasound** |  |  |  |  |  |  |  |  |  |  |  |  |  |  |
| Gallbladder wall irregularities | no | 258 | 64.3 | 139 | 57.7 | 119 | 74.4 | 1 |  |  |  |  |  |  |
| n=401^2^ | yes | 143 | 35.7 | 102 | 42.3 | 41 | 25.6 | 2.1 | 1.34-3.21 | 0.001 | 0.013 | 1.3 | 0.85-1.97 |  |
| Gallbladder wall thickened | no | 130 | 32.4 | 61 | 25.3 | 69 | 43.1 | 1 |  |  |  |  |  |  |
| n=401^2^ | yes | 271 | 67.6 | 180 | 74.7 | 91 | 56.9 | 2.2 | 1.40-3.39 | 0.001 | 0.009 | 0.9 | 0.61-1.40 |  |
| Halo in a gallbladder | no | 148 | 36.9 | 76 | 31.5 | 72 | 45.0 | 1 |  |  |  |  |  |  |
| n=401^2^ | yes | 253 | 63.1 | 165 | 68.5 | 88 | 55.0 | 1.8 | 1.25-2.71 | 0.002 | 0.021 | 0.9 | 0.65-1.35 |  |
| Gallbladder stones | no | 353 | 88.1 | 201 | 83.1 | 152 | 95.6 | 1 |  |  |  |  |  |  |
| n=401^2^ | yes | 48 | 11.9 | 41 | 16.9 | 7 | 4.4 | 4.3 | 1.89-9.67 | <0.001 | 0.009 | 2.8 | 1.33-6.04 | 0.007 |
| Increased echogenicity of liver tissue, n=436 | no | 145 | 33.3 | 63 | 23.8 | 82 | 48.0 | 1 |  |  |  |  | | |
|  | yes | 291 | 66.7 | 202 | 76.2 | 89 | 52.1 | 2.9 | 2.00-4.15 | <0.001 | <0.001 | square root of VIF > 2, excluded | | |
| Periductal fibrosis of | 0 | 80 | 18.4 | 21 | 7.9 | 59 | 34.5 |  |  |  |  |  |  |  |
| intrahepatic bile ducts | Grade 1 | 291 | 66.7 | 197 | 74.3 | 94 | 55.0 |  |  |  |  |  |  |  |
| n=436 | Grade 2 | 65 | 14.9 | 47 | 17.7 | 18 | 10.5 | 4.0 | 2.41-6.47 | <0.001 | <0.001 | 1.9 | 1.08-3.46 | 0.026 |
|  |  | **Total, sd** | | ***O. f.* positive, sd** | | ***O. f.* negative, sd** | |  |  |  |  |  |  |  |
| Body Mass Index, n=436 |  | 27.4 ± 7 | | 28.7 ± 6.4 | | 25.5 ± 7.4 | | 1.1 | 1.03-1.12 | 0.001 | 0.012 | 1.0 | 0.95-1.02 |  |
| **Abdominal ultrasound** |  |  | |  | |  | |  |  |  |  |  |  |  |
| Gallbladder length, n=401^2^ |  | 73.0 ± 15.5 | | 76.4 ± 16.6 | | 68.0 ± 12.0 | | 1.0 | 1.02-1.06 | <0.001 | 0.004 | 1.02 | 1.01-1.04 | 0.004 |
| Oblique vertical size, n=436 |  | 143.76 ± 20.7 | | 148.6 ± 18.3 | | 136.5 ± 22.2 | | 1.0 | 1.02-1.04 | <0.001 | <0.001 | 1.01 | 0.997-1.03 |  |

**______________________**

- *O. f.* - *Opisthorchis felineus*

^2^ – For the ultrasound of gallbladder, persons with cholecystectomy in the past were excluded, n=35, sd - standard deviation

1. [↑](#endnote-ref-1)
